# Functional analysis of *Candida albicans* protein kinases identifies Crk1 as a modulator of epithelial cell damage

**DOI:** 10.1128/mbio.01764-26

**Published:** 2026-08-20

**Authors:** Anna Möslinger, Allon Weiner, Sascha Schäuble, Bernardo Ramírez-Zavala, Nadja Jablonowski, Zoltán Cseresnyés, Tim B. Schille, Marc Thilo Figge, Gianni Panagiotou, Joachim Morschhäuser, Lydia Kasper, Mark S. Gresnigt, Stefanie Allert, Bernhard Hube

**Affiliations:** 1 Department of Microbial Pathogenicity Mechanisms, Leibniz Institute for Natural Product Research and Infection Biology–Hans-Knöll-Institute 28406, Jena, Germany; 2 Sorbonne Université, CNRS, Inserm, Centre d’immunologie et des maladies infectieuses, CIMI 27063, Paris, France; 3 Department of Microbiome Dynamics, Leibniz Institute for Natural Product Research and Infection Biology–Hans-Knöll-Institute 28406, Jena, Germany; 4 Institute of Molecular Infection Biology, University of Würzburg 9190, Würzburg, Germany; 5 Department of Applied Systems Biology, Leibniz Institute for Natural Product Research and Infection Biology–Hans-Knöll-Institute 28406, Jena, Germany; 6 Cluster of Excellence Balance of the Microverse, Friedrich Schiller University Jena 9378, Jena, Germany; 7 Faculty of Biological Sciences, Friedrich Schiller University 9378, Jena, Germany; 8 Jena University Hospital 39065, Jena, Germany; 9 Institute of Novel and Emerging Infectious Diseases, Friedrich-Loeffler-Institute, Greifswald, Germany; 10 Junior Research Group Adaptive Pathogenicity Strategies, Leibniz Institute for Natural Product Research and Infection Biology–Hans-Knöll-Institute 28406, Jena, Germany; Instituto Carlos Chagas, Curitiba, Brazil

**Keywords:** *Candida albicans*, epithelial cells, protein kinases, metabolism, pathogenesis

## Abstract

The commensal and pathogenic lifestyles of the opportunistic fungal pathogen *Candida albicans* require complex signaling networks regulated by protein kinases. To investigate the role of *C. albicans* protein kinases at the intestinal epithelial interface, we screened a comprehensive protein kinase deletion library for the capacity of the mutants to damage intestinal epithelial cells (IEC). Mutants showing altered IEC cytotoxicity relative to the wild type were further analyzed for their growth and morphology, focusing on hyper-damaging strains to identify kinases that rather prevent host cell damage. Deletion of *CRK1* caused increased IEC-specific damage, despite slower growth, reduced hyphal length, and reduced adhesion as compared to wild-type cells. While tissue invasion levels and the formation of transcellular tunnels of the *crk1*Δ/Δ mutant were increased, the translocation capacity through the IEC barrier was reduced. Transcriptional and metabolic profiling suggested a role for Crk1 in metabolic adaptation to carbon and nitrogen sources, which was validated by showing that high glucose and amino acids are required for *crk1*Δ/Δ to cause increased IEC damage. Deletion of *CRK1* rendered *C. albicans* more susceptible to cell wall and membrane stressors, but caused higher resistance to a catalase-specific and histidine biosynthesis inhibitor. This phenotypic pattern of medium- and epithelial cell type-specific cytotoxicity displayed by a *C. albicans* protein kinase mutant suggests that Crk1 regulates processes linked to carbon and amino acid metabolism that are relevant to interactions with intestinal epithelial cells.

**IMPORTANCE:**

Microbial signal transduction pathways regulate adaptation to changing environmental conditions and facilitate the success of many microbes during interactions with their hosts. The fungal pathobiont *Candida albicans* exists as a harmless commensal on mucosal surfaces of most humans but can also cause superficial and invasive infections under certain circumstances. Both lifestyles require complex signaling networks, predominantly regulated by protein kinases. The *C. albicans* genome was predicted to encode 108 protein kinases, yet nearly 50% remain uncharacterized. We aimed to dissect the role of *C. albicans* protein kinases during the transition from commensal to pathogen. We showed that multiple protein kinase genes are involved in epithelial cell damage. Particularly, the protein kinase gene Crk1 was of interest because deletion of CRK1 caused increased damage to intestinal epithelial cells under distinct conditions. Our study links Crk1 with regulation of metabolic processes relevant for commensalism and pathogenicity of *C. albicans*.

## INTRODUCTION

The pathogenic fungus *Candida albicans* exists as part of the mycobiota on various mucosal sites, including the oral cavity, the vagina, and the gastrointestinal tract (1–7). However, under certain predisposing conditions, such as microbiota dysbiosis or chemotherapy, *C. albicans* can overgrow and cause superficial or even systemic infection (8). The gastrointestinal tract is the main origin for systemic infections (9–11).

Epithelial cells are the first host cells in contact with *C. albicans*, which act as a barrier and can mount initial immune responses (12–15). During pathogenesis, *C. albicans* adheres to epithelial cells, followed by invasion of the epithelial tissue. *C. albicans* invasion into epithelial cells occurs via two routes: induced endocytosis triggered by hypha-associated factors initiating actin re-organization and uptake of *C. albicans*, and active fungal penetration caused by physical forces of elongating hyphae (13, 16, 17). Invasion of epithelial cells by *C. albicans* hyphae is cell type dependent. While invasion of intestinal microfold (M), oral, and vaginal epithelial cells is due to a combination of active penetration and induced endocytosis, invasion of intestinal enterocytes is mainly caused by active penetration (13–15, 18, 19). Secretion of hypha-associated factors, in particular the peptide toxin candidalysin, into the invasion pocket is essential for epithelial damage (20, 21). However, epithelial cells have developed calcium-dependent repair mechanisms to counteract candidalysin-mediated damage (22). Systemic infections with *C. albicans* are initiated by translocation through the intestinal epithelial barrier. For example, *C. albicans* hyphae can translocate via the transcellular route, a process dependent on the secretion of candidalysin. Alternatively, *C. albicans* can cross intestinal epithelial layers through physically damaged barriers, via a paracellular route or via M cells or Peyer’s patches (14, 19, 23, 24).

One of the most important virulence traits of *C. albicans* is the ability to grow in both yeast and hyphal morphologies (25). While hyphae mediate efficient epithelial invasion, yeast cells facilitate systemic dissemination (26, 27). Furthermore, both morphologies are required for gut colonization (26, 28).

Metabolic flexibility is also an important trait for both lifestyles, commensal growth, and pathogenicity of *C. albicans* (29). For example, the ability to acquire and metabolize diverse carbon and nitrogen sources is required for the colonization of the gastrointestinal tract (29, 30). However, transcriptional regulators involved in either colonization or infection can differ. While the major regulator gene of carbohydrate metabolism, *TYE7*, is required to proliferate in the gastrointestinal tract, it is dispensable during systemic infection (30). Alternatively, Rtg1/3 and Hms1 regulate genes involved in hexose catabolism and are required for both colonization and systemic infection (30). Similarly, the alternative carbon source acetate is abundant in the gastrointestinal niche, and a complete loss of acetate uptake (by deletion of *ATO1-10* and *JEN1-2*) significantly reduced colonization levels of *C. albicans* in mice (31).

Virulence traits such as the yeast-hyphae transition or metabolic flexibility require a complex network of signaling pathways. Protein kinases play a central role in the regulation of nearly all signaling pathways and have been a focus in the search for drug targets of modern pharmacology (32–34). Previous screening of protein kinase deletion libraries and further studies investigated the involvement of protein kinases in stress response (35–38), morphogenesis (33, 34, 39–41), biofilm formation (33), iron limitation (32), and other processes associated with virulence (40–46). While these previous screens and studies cover important virulence traits, not many protein kinases, except those involved in hyphal formation, have been described as regulators of processes leading to or preventing epithelial cell damage.

This study focuses on the role of *C. albicans* protein kinases during interaction of the fungus with human intestinal epithelial cells. For this, we used a protein kinase mutant library with individual mutants lacking one or both alleles of predicted protein kinase genes in *C. albicans* (32) and screened the ability of each mutant to damage intestinal epithelial cells. Mutants with altered epithelial cytotoxicity were validated and further investigated. Collectively, we identified a crucial role for several protein kinases during infection of intestinal epithelial cells, including kinases that negatively and hypha independently impact epithelial damage, and focused on the role of Crk1 due to an unusual pathogenicity pattern of the corresponding mutant.

## RESULTS

### Protein kinases are involved in intestinal epithelial cell cytotoxicity

**Fig 1:**
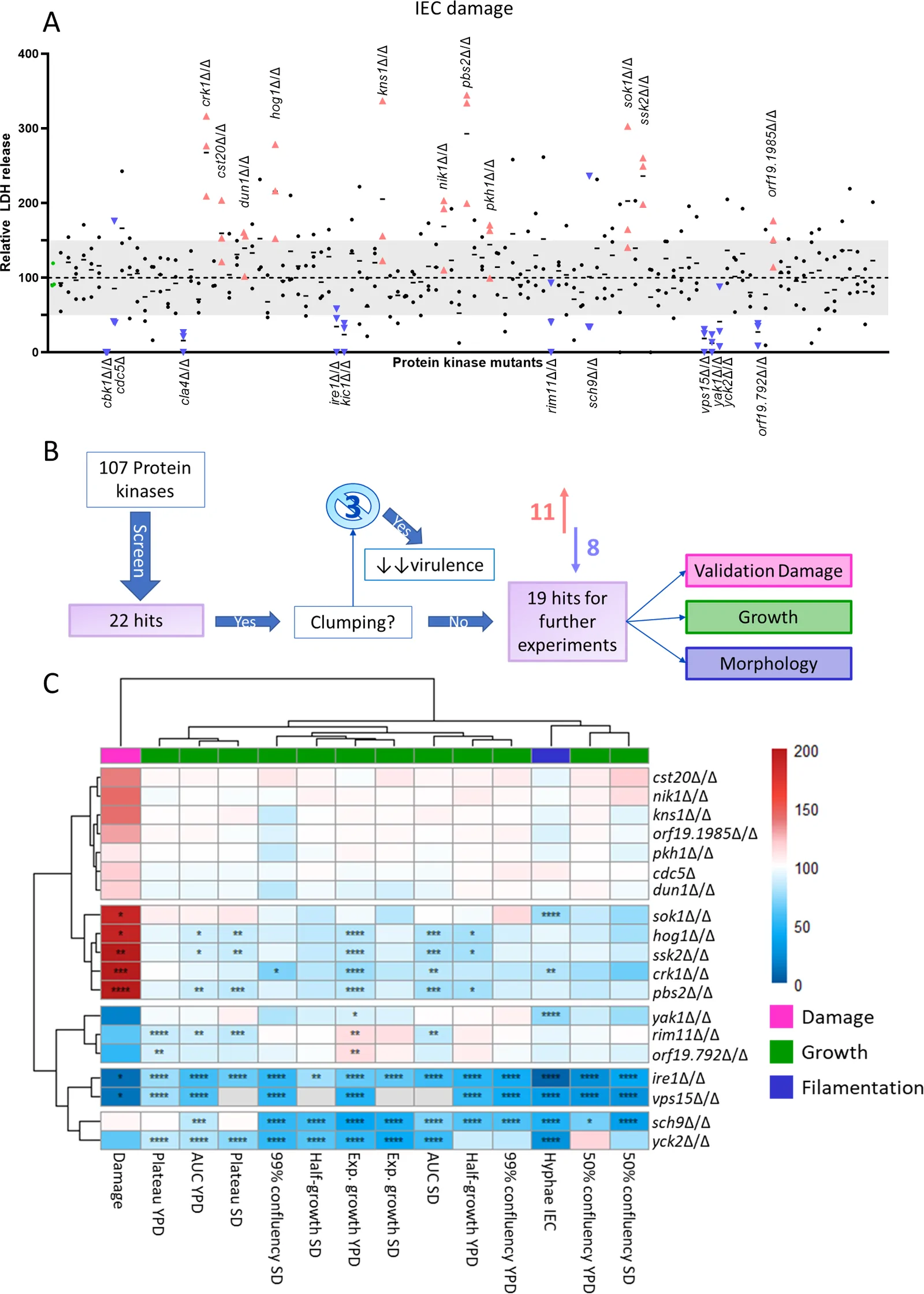
Protein kinases are involved in cytotoxicity, filamentation, and growth. (**A**) Individual protein kinase deletion mutants were assessed for their ability to damage IECs. The LDH release is displayed as the percentage of the wild-type strain (green). Mutants with increased (red) or decreased (blue) IEC cytotoxicity (±50% compared to the wild type) in at least two biological replicates were identified as hits. (**B**) Schematic representation of the selection process and further experiments. (**C**) Validation of the cytotoxicity screen. Data are displayed as the percentage relative to the wild type to show reduced (blue) and increased (red) IEC damage (pink), growth in minimal (SD) or complete (YPD) media at 30°C (plateau, exponential [exp.] growth, and half-growth), and at 37°C and 5% CO_2_ (confluency) (green), and filamentation on IEC at 6 h post-infection at 37°C and 5% CO_2_ in the cell culture medium DMEM (blue). Data were tested for significance using one-way ANOVA and Dunnett’s multiple comparisons test: *P < 0.05, **P < 0.01, ***P < 0.001, ****P < 0.0001.

The *C. albicans* genome was predicted to encode 108 protein kinases (32). To investigate the role of protein kinases in *C. albicans* pathogenicity, we screened a library containing individual (85 homozygous and 22 heterozygous) *C. albicans* mutants (32) for their ability to damage differentiated intestinal epithelial cells (IECs) (47). Cytotoxicity was quantified by measuring the release of lactate dehydrogenase (LDH) by IECs. Mutants displaying an altered IEC cytotoxicity (±50% of the wild-type strain SC5314) in at least two replicates were further investigated (Fig. 1A and B). We identified 22 mutants with altered IEC cytotoxicity, suggesting a role of these protein kinases in pathogenicity or commensal-like stage. In addition to reduced IEC cytotoxicity, deletion of *CBK1*, *KIC1*, and *CLA4* caused aggregation of yeast cells prior to the infection (Fig. 1B; Fig. S1A). Cbk1 and Kic1 are part of the regulation of Ace2 and morphogenesis (RAM) pathway, which has been previously linked to the regulation of various cellular processes, including cell separation and cell cycle (48–50). Cla4 is part of the Cdc42 pathway and has been shown to be involved in septin ring localization (51, 52). Although the corresponding mutants (*cbk1*Δ/Δ, *kic1*Δ/Δ, and *cla4*Δ/Δ) displayed reduced cytotoxicity, we excluded these from subsequent experiments due to the cell aggregation phenotypes.

### Mutants lacking *CRK1* cause increased or wild-type-like IEC cytotoxicity despite shorter hyphae and slower growth

For the remaining 19 mutants exhibiting altered IEC cytotoxicity (11 or eight mutants with increased or decreased cytotoxicity, respectively), we validated their ability to damage IECs (Fig. 1C; Fig. S1B). Seven mutants caused statistically significantly increased (*pbs2*Δ/Δ, *crk1*Δ/Δ, *ssk2*Δ/Δ, *sok1*Δ/Δ, and *hog1*Δ/Δ) or decreased (*vps15*Δ/Δ, and *ire1*Δ/Δ) IEC cytotoxicity compared to the wild type, suggesting a role for these kinases in colonization or infection, respectively. Interestingly, three of these genes (*HOG1, PBS2,* and *SSK2*) are part of the *HOG1* pathway, best known for its involvement in osmotic stress response (53–55). Moreover, its role in colonization and virulence has been thoroughly studied (36, 37, 41, 43–46, 56, 57).

Subsequently, we investigated the hyphal length of the mutants identified in the IEC cytotoxicity screen. Seven (*crk1*Δ/Δ, *sok1*Δ/Δ, *yak1*Δ/Δ, *sch9*Δ/Δ, *vps15*Δ/Δ, *yck2*Δ/Δ, and *ire1*Δ/Δ) out of the 19 mutants tested showed statistically significantly reduced hyphal length on IECs compared to the wild type in Dulbecco’s modified Eagle medium (DMEM) (same as for the damage assays) at 6 h post-infection at 37°C and 5% CO_2_ (Fig. 1C; Fig. S1C and D). While most of these mutants still formed hyphae similar to the wild type, albeit shorter, *ire1*Δ/Δ did not filament, and *yck2*Δ/Δ and *vps15*Δ/Δ showed short hyphae clumping together (Fig. S1D). Of note, *crk1*Δ/Δ and *sok1*Δ/Δ caused increased IEC damage compared to the wild type despite shorter hyphae (Fig. 1C; Fig. S1B through D).

Finally, the 19 mutants identified for altered cytotoxicity were tested for their ability to grow in minimal (SD) or rich (YPD) media in yeast morphology (30°C) or under hypha-inducing conditions (37°C). To compare different stages of yeast growth, the stationary phase (plateau), exponential growth, the time to reach 50% of the stationary phase (half-growth), and the area under the curve (AUC) were investigated (Fig. 1C). Four mutants (*ire1*Δ/Δ, *vps15*Δ/Δ, *yck2*Δ/Δ, and *rim11*Δ/Δ) reached the stationary phase at a significantly lower OD_600_ compared to the wild-type strain in both media, and *orf19.792*Δ/Δ reached a lower OD_600_ in YPD (Fig. 1C; Fig. S2A). A mutant lacking *VPS15* was unable to grow in minimal media; therefore, the different growth stages could not be calculated (Fig. 1C; Fig. S2A). Additionally, five mutants (*crk1*Δ/Δ, *sch9*Δ/Δ, *ssk2*Δ/Δ, *pbs2*Δ/Δ, and *hog1*Δ/Δ) grew more slowly compared to the wild-type strain but reached the stationary phase at a similar OD_600_ compared to the wild type (Fig. 1C; Fig. S2B). The total growth (AUC) was significantly reduced for eight mutants (*ire1*Δ/Δ, *vps15*Δ/Δ, *yck2*Δ/Δ, *rim11*Δ/Δ, *sch9*Δ/Δ, *ssk2*Δ/Δ, *pbs2*Δ/Δ, and *hog1*Δ/Δ) in both media, whereas the *crk1*Δ/Δ showed significantly reduced total growth only in minimal media (Fig. 1C). For growth in hypha-inducing conditions, the time to reach 50% or 99% confluency of the well was assessed. Only three mutants (*ire1*Δ/Δ, *vps15*Δ/Δ, and *sch9*Δ/Δ) showed a significantly longer time to reach 50% and 99% confluency in complete media, and five mutants (*ire1*Δ/Δ, *vps15*Δ/Δ, *sch9*Δ/Δ, *yck2*Δ/Δ [only 99%], and *crk1*Δ/Δ [only 99%]) showed a significantly longer time to reach 50% or 99% confluency in minimal media.

In summary, we identified several protein kinase genes involved in growth, filamentation, and capacity to damage IECs. Two protein kinase genes (*VPS15* and *IRE1*) were involved in filamentation, growth, and cytotoxicity. Mutants lacking these genes showed extensive filamentation and growth defects and, as a result, also caused little epithelial damage in comparison to the wild type. These results confirm the previously proposed importance of *C. albicans* hyphae formation in damaging the epithelial layer (14, 15). However, we also identified two mutants with decreased hyphal length and slower growth, showing similar (*sch*9Δ/Δ) or increased (*crk1*Δ/Δ) IEC cytotoxicity compared to the wild type. This suggests that mutants with shorter hyphae can cause similar or even increased damage compared to the wild type.

Sch9 has a central role in different nutrient signaling pathways (58–60). For example, in response to glucose or nitrogen sources, Sch9 is regulated by PKA or Tor1, respectively (59), whereas in response to lipids, Pkh1-2 regulates fungal CO_2_ sensing through Sch9 (60). However, in our screen, the heterozygous mutant *tor1*Δ and homozygous mutant *pkh1*Δ/Δ did not cause altered IEC cytotoxicity compared to the wild type. This suggests that the reduced filamentation, slower growth, and wild-type-like cytotoxicity observed in *sch*9Δ/Δ are potentially regulated via different pathways. Since *crk1*Δ/Δ and *sch9*Δ/Δ show similar phenotypes, we investigated whether Crk1 is, similar to Sch9, also involved in the TORC1 pathway. Therefore, the growth of *C. albicans* strains (SC5314, *crk1*Δ/Δ, *sch*9Δ/Δ, and *tor1*Δ) was assessed in rich media in the presence or absence of rapamycin over 72 h (Fig. S2C). While growth of *sch*9Δ/Δ and *tor1*Δ was significantly reduced in the presence of rapamycin in comparison to the wild type, *crk1*Δ/Δ showed similar growth compared to the wild type. This suggests that Crk1 is not involved in the TORC1 pathway and that the observed phenotypes are caused by dysfunctions of other pathways. Therefore, we focused on the characterization of Crk1 and its interaction with epithelial cells.

### The increased cytotoxicity caused by *crk1*Δ/Δ is cell type specific

**Fig 2:**
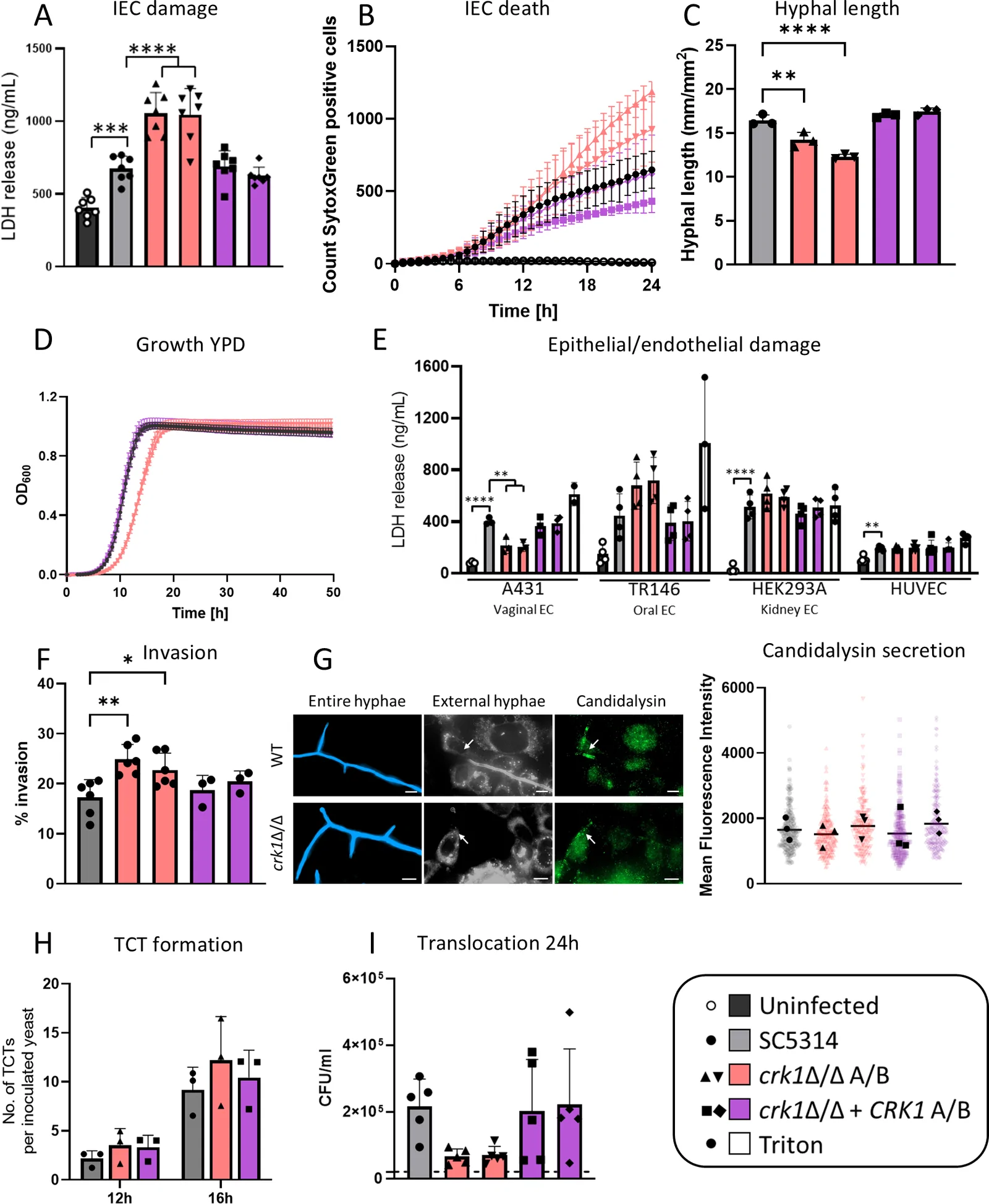
Deletion of *CRK1* causes increased IEC damage, and this is cell type specific. (**A**) LDH release was measured 24 h post infection. (**B**) Dead epithelial cells were stained by SYTOX Green nucleic acid stain, and images were acquired every 45 min to quantify host cell death. (**C**) Hyphal length was quantified after 6 h at 37°C and 5% CO_2_ in the cell culture medium DMEM. (**D**) Growth in rich medium was monitored by measuring the OD_600_ every 30 min over 50 h. Error bars represent the mean ± standard deviation. (**E**) Damage of epithelial (EC) and endothelial cells was quantified by measuring the release of LDH after 24 h. (**F**) Invasion is defined as the number of hyphae with invasive parts relative to the number of all hyphae (external + internal) at 8 h post-infection. (**G**) Release of the peptide toxin candidalysin was assessed by measuring the invasive part of hyphae and quantifying the mean fluorescence intensity of the candidalysin antibody. Arrows indicate the invasive parts of hyphae and the candidalysin staining. (**H**) The number of transcellular tunnels (TCT) was counted relative to the number of inoculated yeasts per field of view. (**I**) Colony-forming units (CFUs) per mL were counted and calculated after 24 h relative to the initial inoculum. The dotted line represents the initial inoculum. Bars represent the mean ± standard deviation, and dots represent the mean of each biological replicate. Data were tested for significance using one-way ANOVA and Dunnett’s multiple comparisons test (**A, C, E, F–I**). Statistical tests were carried out separately for each cell line (**E**) and each time point (**H**). *P < 0.05, **P < 0.01, ***P < 0.001, ****P < 0.0001.

To prove that the observed phenotypes are in fact caused by a deletion of *CRK1*, we re-inserted *CRK1* and tested two independently generated deletion (*crk1*Δ/Δ A/B) and revertant (*crk1*Δ/Δ + *CRK1* A/B) strains. Both deletion mutants exhibited decreased hyphal length, slower growth in complete and minimal media, and increased IEC cytotoxicity compared to the wild type, whereas the revertant strains behaved similarly to the wild type (Fig. 2A through D; Fig. S3A and B). In addition to fungus-induced cytotoxicity and background levels of extracellular LDH release of uninfected IECs, a high-damage control (0.25% Triton) was added, and LDH release of uninfected IECs corresponded to around 14%, the wild-type and revertant strains to around 23%, and the *CRK1* deletion mutants to around 45% of the high control. To validate the increased damage, we also monitored the dynamics of IEC death using the nucleic acid stain SYTOX Green in live-cell imaging. Similar to the LDH assay, both *crk1*Δ/Δ mutants caused increased epithelial cell death compared to the wild-type and revertant cells starting from 13 h post-infection (Fig. 2B).

To test whether the increased IEC cytotoxicity is also observed in other epithelial and endothelial cells, we investigated the cytotoxicity against vaginal, oral, and kidney epithelial cell lines, as well as primary human umbilical cord endothelial cells (HUVEC) when infected with *crk1*Δ/Δ by measuring LDH release 24 h post-infection. While *crk1*Δ/Δ caused slightly increased cytotoxicity of oral and kidney epithelial cells compared to the wild-type and revertant strains, HUVEC cells were damaged to a similar extent by all strains tested (Fig. 2E). Interestingly, *crk1*Δ/Δ caused significantly reduced vaginal epithelial cytotoxicity compared to the wild-type and revertant strains (Fig. 2E). These results indicate that the observed increased cytotoxicity of *crk1*Δ/Δ is cell type specific.

### Lack of *CRK1* causes increased intestinal epithelial invasion and transcellular tunnels but decreased translocation

Since the increased cytotoxicity of *crk1*Δ/Δ is IEC specific, we further investigated the interaction between *crk1*Δ/Δ and IEC by measuring adherence, invasion, and translocation potential. While *C. albicans* lacking *CRK1* showed slightly decreased adhesion to IECs and plastic at 1 h post-infection (Fig. S3B), invasion of IECs was significantly increased compared to the wild-type and revertant strains at 8 h post-infection (Fig. 2F). However, the percentage of the invasive part of the hyphae compared to the entire fungal mass was similar between all strains tested (Fig. S3C). The delivery of the *C. albicans* toxin candidalysin into the invasion pocket is required for the full damage potential of *C. albicans* (20). At 8 h post-infection, all strains tested secreted similar levels of candidalysin (Fig. 2G). Furthermore, at this time point, low levels of epithelial cell death were detected, and there was no difference between the tested strains (Fig. 2B). When *C. albicans* hyphae invade intestinal epithelial cells, the invading hyphae are surrounded by an epithelial membrane and form transcellular tunnels that can traverse several host cells in sequence (61). To test invasion characteristics at later time points, we stained the epithelial membrane during live-cell imaging and counted the number of transcellular tunnels formed per inoculated yeast (Fig. 2H). In this assay, *crk1*Δ/Δ formed more transcellular tunnels compared to the wild type at 12 and 16 h post-infection (Fig. 2H). Since invasion of IECs is primarily caused by active fungal penetration (14, 18), we investigated whether increased IEC invasion by *crk1*Δ/Δ was due to increased physical forces. Therefore, the ability of the *C. albicans* strains to invade different concentrations of agar was assessed. While the wild-type and revertant strains fully invaded 1.5%, 2%, 3%, and 4% of agar after 2 days, *crk1*Δ/Δ was only able to invade all agar concentrations after 4 days (Fig. S3D), suggesting that Crk1 is required to maintain full physical force capacity of hyphae.

Finally, we investigated whether the observed increased epithelial invasion (Fig. 2F and H) is associated with increased translocation through epithelial layers. Therefore, colony-forming units (CFUs) of translocated *crk1*Δ/Δ cells were quantified at 16 and 24 h post-infection. Less translocation of *crk1*Δ/Δ mutant cells through the epithelial barrier was observed (about 30% of wild type) compared to the wild type at both time points (Fig. 2I; Fig. S3E). Next, we checked whether the decreased translocation is due to increased sensitivity or resistance to zymolyase, which is used in the translocation assay to detach translocated hyphae from the membrane barrier (14). However, the survival and growth after zymolyase treatment were similar in all strains tested (Fig. S2F).

In conclusion, Crk1 plays a role in IEC invasion, formation of transcellular tunnels, and translocation; however, the mechanisms leading to increased cytotoxicity remain unclear.

### Crk1 is involved in glucose and amino acid metabolism

**Fig 3:**
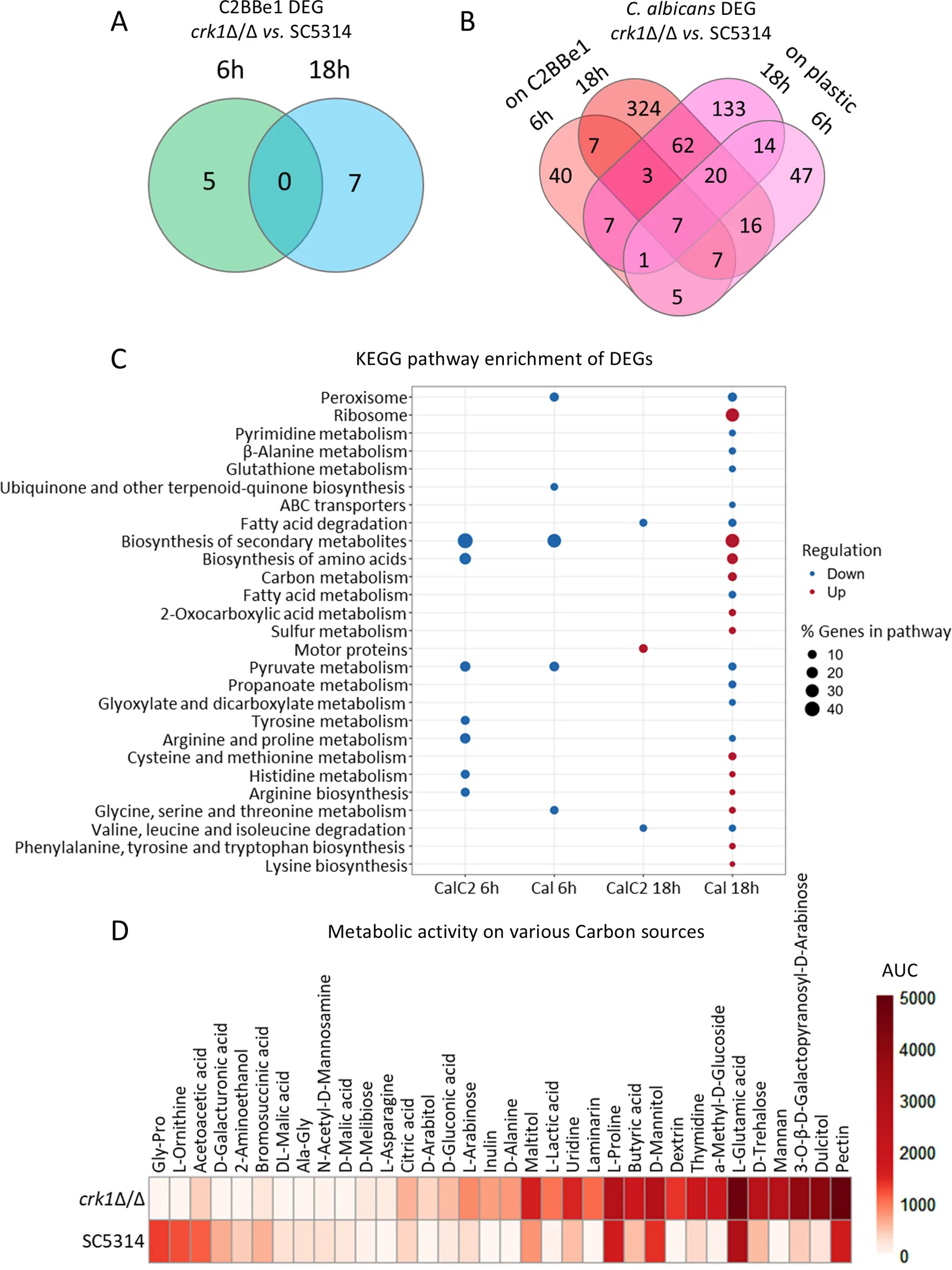
*CRK1* is involved in amino acid and carbon metabolism. (**A**) Venn diagram depicting significantly differentially expressed genes (DEG; log2 fold change ± 1, *P* < 0.05; comparison *crk1*Δ/Δ vs SC5314) in C2BBe1 after *C. albicans* infection at 6 and 18 h. (**B**) Venn diagram depicting significantly differentially expressed genes (DEG; log2 fold change ± 1, *P* < 0.05; comparison *crk1*Δ/Δ vs SC5314) in *C. albicans* strains on C2BBe1 or plastic at 6 and 18 h. (**C**) KEGG network enrichment analysis on *C. albicans* significantly differentially expressed genes (log2 fold change ± 0.5, *P* < 0.05) in the *crk1*Δ/Δ mutant compared to the wild type on C2BBe1 (CalC2) or plastic (Cal). (**D**) Carbon metabolites showing different metabolic activity of wild type (SC5314) and *crk1*Δ/Δ in Biolog phenotypic microarray analysis.

To gain more insights into the processes causing increased IEC cytotoxicity by *crk1*Δ/Δ, we performed transcriptional profiling of differentiated IECs (C2BBe1), *C. albicans* wild type (SC5314), or *crk1*Δ/Δ A cells at 6 and 18 h post-infection (Tables S1 and S2). Overall, very few IEC genes were significantly up- or downregulated when infected with *crk1*Δ/Δ compared to SC5314 (Fig. 3A), suggesting a more general transcriptional response of epithelial cells to *C. albicans* not affected by Crk1. In contrast, the transcriptional profiles of *crk1*Δ/Δ compared to the wild type in the presence or absence of IECs included multiple differentially expressed genes (DEGs), particularly at 18 h post-infection (Fig. 3B; Table S2). KEGG enrichment analysis showed that many of these genes are involved in the metabolism of carbon sources and various amino acids (Fig. 3C).

### Crk1 is involved in the regulation of the tricarboxylic acid (TCA) cycle

To further study the role of Crk1 in *C. albicans* metabolism, we used Biolog phenotypic microarrays to investigate the metabolic activity on 190 different carbon and 380 nitrogen (containing nitrogen, amino acids, and di- and tripeptides) sources. To assess the metabolic activity of *crk1*Δ/Δ and the wild type on various carbon sources, we compared the AUC obtained from measuring the metabolic activity (OD_590_) every 20 min over 48 h (Table S3). Metabolic activity was defined as having an AUC difference of more than 50 compared with the negative control (Table S3). In contrast to the wild type, *crk1*Δ/Δ showed metabolic activity on L-asparagine, D-arabitol, inulin, D-alanine, L-lactic acid, laminarin, dextrin, α-methyl-D-glucoside, and mannan, whereas only the wild type was metabolically active on Gly-Pro, L-ornithine, D-galacturonic, 2-aminoethanol, DL-malic acid, D-malic acid, Ala-Gly, and N-acetyl-D-mannosamine. Most carbon sources that led to altered AUC between *crk1*Δ/Δ and the wild type are involved in or feed into the TCA cycle (Fig. 3D). Since the TCA cycle is linked to amino acid biosynthesis, we assessed the growth of the wild type and *crk1*Δ/Δ on TCA cycle intermediates over 48 h (Fig. S4C; Table S3). While growth on glucose and pyruvate was similar, *crk1*Δ/Δ showed earlier and increased growth when malate, succinate, α-ketoglutarate, or sodium lactate was provided as the sole carbon source. In contrast, growth on citrate was slightly reduced in *crk1*Δ/Δ compared to the wild type (Fig. S4C). Of note, *crk1*Δ/Δ showed increased metabolic activity on the negative control of the nitrogen source plates (only containing glucose, potassium phosphate monobasic anhydrous, and sodium sulfate in the media) compared to the wild type (Fig. S4A). Therefore, we did not include these plates for our analysis. However, it suggests that Crk1 may be involved in glucose uptake or utilization. Therefore, we measured glucose concentrations in the supernatant of wild type or *crk1*Δ/Δ A cells grown in minimal media containing 20 mM glucose (slightly lower than DMEM levels). There was a significant increase in glucose concentration in media used to culture *crk1*Δ/Δ compared to wild type after 5, 6, and 7 h of incubation at 37°C (Fig. S4B), despite similar growth in both strains tested. This suggests that Crk1 is important for glucose uptake and regulates metabolism of TCA cycle intermediates that are also important for amino acid biosynthesis.

### Amino acids and high-glucose levels are required for *crk1*Δ/Δ-associated increased IEC cytotoxicity

**Fig 4:**
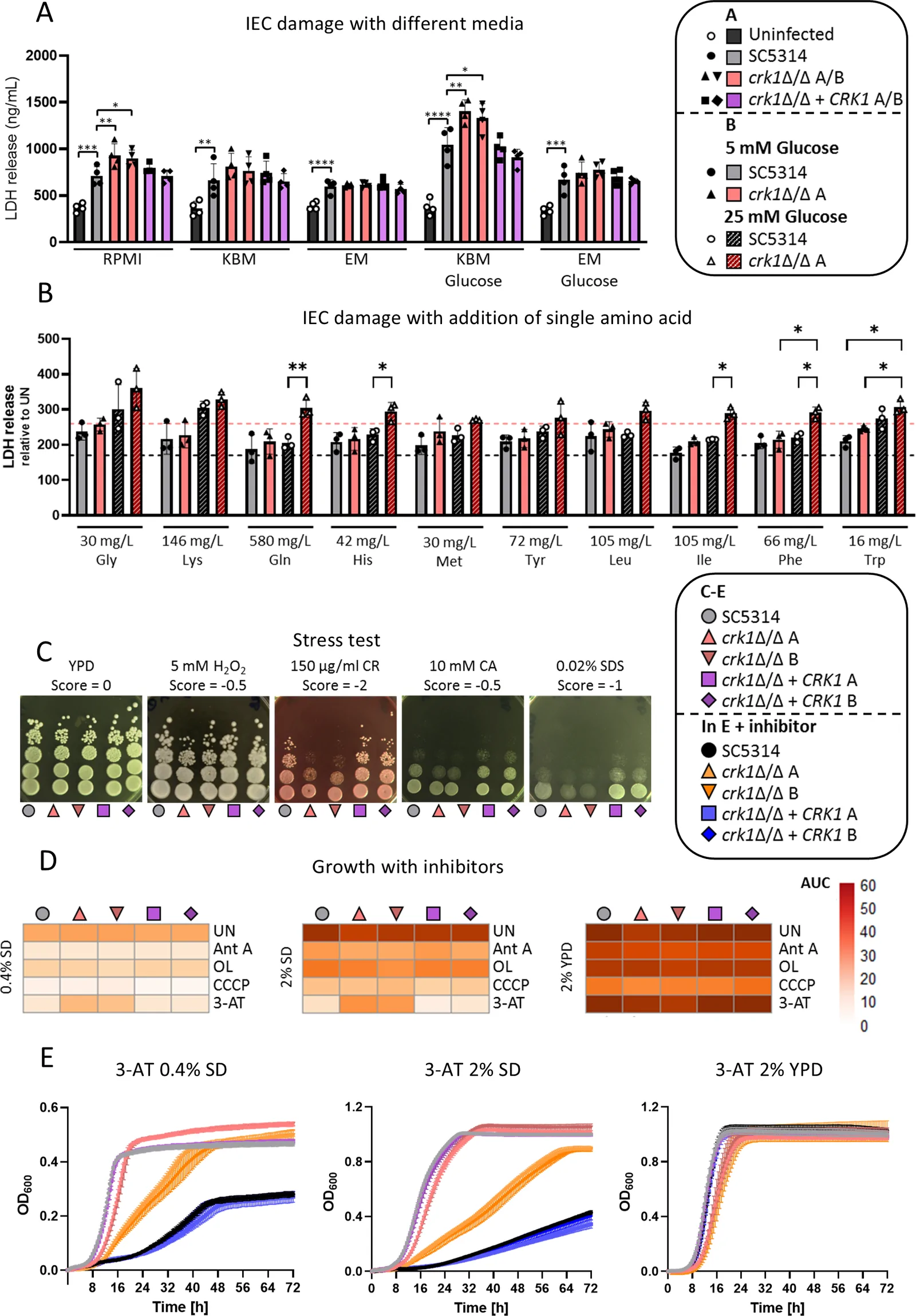
The increased IEC cytotoxicity of *crk1*Δ/Δ is media-dependent. (**A**) LDH release was measured in the supernatant at 24 h post-infection in different cell culture media (DMEM, RPMI, KBM) and EM. KBM and EM were tested with low- (~12 mM or 5 mM, respectively) or high-glucose (~25 mM proxy to DMEM) levels. Bars represent the mean ± standard deviation, and dots represent the mean of each biological replicate. Data were tested for significance using one-way ANOVA and Dunnett’s multiple comparisons test. Statistical test was carried out separately for each cell line (**E**). (**B**) Single amino acids were added to low- or high-glucose EM in the same concentrations as in DMEM, and the LDH release was measured in the supernatant at 24 h post-infection and shown relative to the uninfected control. The dotted lines represent the LDH release of wild type (black) and *crk1*Δ/Δ (red) in DMEM. Bars represent the mean ± standard deviation, and dots represent the mean of each biological replicate. Data were tested for significance using two-way ANOVA and Tukey’s multiple comparisons test. (**C**) Spot dilution assay in the presence of temperature, osmotic, oxidative, cell wall, or membrane stressors. The score written corresponds to the growth difference between the *crk1*Δ/Δ mutant and the wild type. (**D**) Heatmap showing the AUC for the growth curves in the presence of 100 μM 3-AT (**E**), 3 μM antimycin-A (Fig. S5B), 10 μM oligomycin (Fig. S5C), and 20 μM CCCP (Fig. S5D) in minimal media (SD; 0.4% or 2% glucose), or rich media (YPD, 2% glucose). Error bars represent the mean ± standard deviation. *P < 0.05, **P < 0.01, ***P < 0.001, ****P < 0.0001.

Since Crk1 seems to be involved in carbon, including glucose, and amino acid metabolism, we tested whether the increased IEC cytotoxicity in DMEM (containing 25 mM glucose and 15 canonical amino acids) is also observed when using different media for infection, i.e., RPMI (containing ~11 mM glucose and 19 canonical amino acids), keratinocyte basal medium (KBM; containing ~12 mM glucose and 20 canonical amino acids), and EM (containing 5 mM glucose and no amino acids) (62). The formulation of KBM is not published; however, the glucose concentration was measured, and amino acid composition was determined by using previously acquired metabolomic data (63). While *crk1*Δ/Δ caused significantly increased IEC damage in RPMI media compared to the wild type, the damage was similar between all tested strains in KBM and EM (Fig. 4A). Addition of 12 mM glucose to KBM (proxy to DMEM levels) increased the cytotoxicity of all strains tested, and in comparison to the wild type, *crk1*Δ/Δ caused increased IEC cytotoxicity (Fig. 4A). However, higher glucose concentrations (25 mM) in EM only slightly increased the cytotoxicity of all strains, and no differences were observed between wild type and *crk1*Δ/Δ (Fig. 4A). To investigate whether specific amino acids are required for *crk1*Δ/Δ to cause increased IEC cytotoxicity, 5% non-essential amino acids (NEAAs) or an amino acid mix (containing glycine, lysine, phenylalanine, threonine, tryptophan, valine, glutamine, leucine, and isoleucine) were added to EM with low (5 mM) or high (25 mM) glucose concentration (Fig. S4E). These amino acids were chosen because they are present in DMEM with a concentration of ≥0.4 mM. While the addition of NEAAs caused similar IEC cytotoxicity of wild type and *crk1*Δ/Δ, addition of the amino acid mix caused increased IEC cytotoxicity by *crk1*Δ/Δ compared to the wild type under both low- and high-glucose conditions (Fig. S4D). To further characterize which amino acids are involved in increased IEC cytotoxicity by *crk1*Δ/Δ, the amino acid mix was further separated into large neutral amino acids (phenylalanine, threonine, tryptophan, valine, leucine, isoleucine; cocktails I, III, IV, V) and the others (glycine, lysine, glutamine; cocktail II). The remaining large neutral amino acids (histidine, methionine, tyrosine; cocktail VI) were also added. The IEC cytotoxicity was increased in *crk1*Δ/Δ compared to the wild type when either cocktail II (glycine, lysine, glutamine) and cocktail VI (histidine, methionine, tyrosine) were added to low- and high-glucose EM, or cocktail I (phenylalanine, threonine, tryptophan, valine, leucine, isoleucine), cocktail IV (phenylalanine, tryptophan), or cocktail V (leucine, isoleucine) were added to high-glucose EM (Fig. S4D). There was no difference between *crk1*Δ/Δ and the wild-type strain when cocktail III (threonine, valine) was added, and therefore, these amino acids were not used for subsequent experiments. As a next step, single amino acids were added to low- or high-glucose EM to further investigate whether specific amino acids are required for the increased cytotoxicity caused by *crk1*Δ/Δ compared to wild type. The presence of glutamine, histidine, isoleucine, phenylalanine, or tryptophan caused significantly increased IEC cytotoxicity in the *crk1*Δ/Δ mutant compared to the wild-type strain (Fig. 4B). This shows that increased IEC cytotoxicity depends not only on the host cell type but also on the culture media. This suggests that Crk1 plays a role in amino acid or glucose metabolism, and this altered metabolism may be involved in causing increased IEC cytotoxicity.

### Crk1 is involved in response to cell wall stress

Since an altered metabolism can be linked to cellular stress responses (29), the response of *crk1*Δ/Δ to temperature, osmotic, oxidative, cell wall, and membrane stressors was tested (Fig. 4C; Fig. S5A). While all strains grew similarly in response to elevated temperature (37°C and 42°C), osmotic (1 M NaCl and 0.6 M KCl), and oxidative (1, 2.5, and 5 mM tert-butyl hydroperoxide [t-BOOH]) stressors, the *crk1*Δ/Δ mutants were more sensitive to cell wall (50, 100, and 150 µg/mL Congo red, and 5, 10, and 15 mM caffeine), membrane (0.01 and 0.02% SDS), and, to a lesser degree, oxidative (2.5 and 5 mM H_2_O_2_) stressors compared to the wild type.

### Deletion of *CRK1* causes increased resistance to catalase and histidine biosynthesis inhibitor

*C. albicans* mitochondria are involved in various non-respiratory processes, including virulence, cell wall biogenesis, and stress response (64), whereas *C. albicans* catalase is one of the main responders to oxidative stress and closely interacts with the mitochondria (65–68). Therefore, we tested the wild type, *CRK1* deletion, and revertant strains for their ability to grow in the presence of various mitochondrial inhibitors, i.e., antimycin A (inhibits electron transport chain), oligomycin (inhibitor of ATP synthase), carbonyl cyanide m-chlorophenylhydrazone (CCCP, protonophore that uncouples oxidative phosphorylation), and the catalase and His3 inhibitor 3-amino-1,2,4-triazole (3-AT). Similar to non-inhibitor-containing medium, *crk1*Δ/Δ also showed slower growth in the presence of oligomycin and antimycin A compared to the wild type (Fig. 4D; Fig. S5B and C). In the presence of CCCP, the growth of *crk1*Δ/Δ was slightly increased in comparison to the wild type (Fig. S5D). Furthermore, the *crk1*Δ/Δ mutant grew slower but nearly reached a similar stationary phase as untreated cells in the presence of 3-AT in minimal media, whereas the wild-type and *CRK1* revertant strains showed reduced growth (Fig. 4E). This could suggest increased catalase or His3 activity in *crk1*Δ/Δ. Thus, Crk1 may be involved in histidine biosynthesis (through His3) or in the breakdown of reactive oxygen species (ROS) or H_2_O_2_ into water and oxygen (through catalase activity). However, growth in the presence of H_2_O_2_ was reduced (Fig. 4C; Fig. S5A). To test whether morphology plays a role in the response to H_2_O_2_, we grew the wild type, *CRK1* deletion, and revertant strains for 8 h at 37°C with 5% CO_2_ in DMEM (hyphae) or minimal media (SD; mixture of yeast and pseudohyphae) (Fig. S6). After treatment with or without 0.5 mM or 1 mM H_2_O_2_, cells were imaged over 16 h, and the confluency of each well was analyzed. In general, there was more growth of all strains observed in minimal media compared to DMEM in both untreated and H_2_O_2_-treated cells (Fig. S6A). After H_2_O_2_ treatment, the *crk1*Δ/Δ mutants grew slightly more compared to the wild-type and revertant strains in DMEM with H_2_O_2_ treatment, and all strains grew similarly in SD media in response to H_2_O_2_ treatment (Fig. S6B). This suggests the importance of media and morphology in the response to stress and that deletion of *CRK1* may result in increased catalase activity or an amino acid starvation response.

## DISCUSSION

Protein kinases of *C. albicans* regulate multiple processes during commensalism and pathogenicity. Our cytotoxicity screen using a protein kinase deletion mutant library in the SC5314 background (32) and additional validation experiments identified a number of protein kinases associated with *C. albicans*-mediated intestinal epithelial damage. Here, we identified protein kinase mutants showing a hyper-damaging phenotype: deletion of five protein kinase genes individually (*HOG1, PBS2, SSK2, CRK1,* and *SOK1*) caused significantly increased IEC damage. Surprisingly, four corresponding mutants showed both increased cytotoxicity and either slower growth or reduced filamentation, whereas deletion of *CRK1* caused both slower growth and shorter hyphal length (Fig. 1C; Fig. S1 and S2). We focused our study on the phenomenon that epithelial cells can be more damaged by mutant cells with growth defects and shorter hyphae compared to wild-type cells and the potential involvement of Crk1. To dissect this phenomenon, we systematically investigated virulence traits, stress resistance, and metabolic activities of *crk1*Δ/Δ in the context of epithelial cells.

Crk1 (Cdc2-related kinase 1) belongs to the cyclin-dependent kinase-related family of serine/threonine protein kinases. Cyclin-dependent kinases have been linked to *C. albicans* morphogenesis (69–72), metabolism and respiration (73), and antifungal treatment (74, 75). While an early study showed that Crk1 is required for hypha formation in defined media (Lee’s medium and serum), and for virulence during systemic infections of mice (40), this study did not investigate interactions with epithelial cells and thus did not observe an enhanced damage capacity of *crk1*Δ/Δ toward epithelial cells. In contrast, the used *in vivo* infection model included direct intravenous injection of a *crk1*Δ/Δ mutant in immunocompetent mice (40).

As the gastrointestinal *C. albicans* population is the main origin for systemic disease (9–11), the intestinal epithelium is an important barrier to prevent systemic infections with this fungus. Accordingly, contact of *C. albicans* with this barrier, followed by invasion and translocation are central events for early stages of invasive infections. Therefore, numerous studies have focused on the interaction of *C. albicans* with intestinal enterocytes (14, 18, 19, 47, 63). In this study, a mixture of differentiated enterocytes and goblet-like cells (ratio 70:30) (47) or undifferentiated or differentiated enterocytes were used to study the role of protein kinases in these critical steps. While deletion of *CRK1* caused significantly increased IEC damage compared to the wild-type strain, oral and kidney epithelial cells and HUVEC were similarly or only slightly more damaged. In contrast, vaginal epithelial cells were significantly less damaged by *crk1*Δ/Δ (Fig. 2A/E), indicating a host cell-specific phenotype.

Invasion of *crk1*Δ/Δ hyphae into ICEs was significantly increased at 8 h, and higher levels of *crk1*Δ/Δ transcellular tunnels were monitored at later time points compared to the wild type (Fig. 2F–H). Invasion by wild-type hyphae and transcellular tunnels within IECs were only associated with damage at later time points (61), likely due to epithelial repair mechanisms (22). Invasion of *C. albicans* hyphae into epithelial cells can occur via two distinct mechanisms—active penetration driven by hyphal elongation and physical forces, and induced endocytosis of epithelial cells triggered by hypha-associated invasins (13). While vaginal, oral, and kidney epithelial cells and HUVEC are invaded via both mechanisms, invasion of intestinal enterocytes occurs primarily by active penetration (13, 14, 18, 19). Therefore, we investigated physical forces of *crk1*Δ/Δ hyphae by monitoring invasion into agar. Agar invasion by *crk1*Δ/Δ cells was reduced compared to wild-type cells, suggesting that the observed increase of invasion by *crk1*Δ/Δ cells into IECs is due to mechanisms non-dependent on physical forces.

Invasion of epithelial cells by *C. albicans* is linked to epithelial repair (22) and associated with the creation of an invasion pocket (76). Secretion of the peptide toxin candidalysin into the invasion pocket is essential for full *C. albicans*-induced epithelial damage (20). Candidalysin secretion was similar between *crk1*Δ/Δ and the wild type at 8 h post-infection (Fig. 2G). However, expression of *ECE1*, encoding candidalysin, in *crk1*Δ/Δ was significantly increased after 18 h on IECs compared to the wild type (Table S2).

Therefore, increased invasion and transcriptional levels of *ECE1* may contribute to the cell-type-specific increased cytotoxicity caused by the *crk1*Δ/Δ mutant. The increased cytotoxicity caused by the *crk1*Δ/Δ mutant required high glucose and the presence of even a single amino acid, i.e., glutamine, histidine, isoleucine, phenylalanine, or tryptophan (Fig. 4A). Previously, genes involved in carbon and nitrogen utilization have been shown to be upregulated in *C. albicans* infection of oral and liver tissue and blood (28, 76, 77). In this study, KEGG analyses showed that genes significantly differently expressed in *crk1*Δ/Δ compared to the wild type were related to carbon and amino acid metabolism (Fig. 3C). Similarly, *crk1*Δ/Δ showed increased growth on the TCA intermediates, α-ketoglutarate, succinate, and malate as a sole carbon source and complex carbon sources (Fig. 3D; Fig. S4C), and the dicarboxylic plasma membrane acid transporter Jen2 (78) was significantly upregulated in *crk1*Δ/Δ after 18 h on IECs (Table S2). In contrast, the growth of *crk1*Δ/Δ was reduced on citrate, and expression of *EED1* (encoding epithelial escape and dissemination 1, Eed1) (76) was significantly downregulated after 18 h on IECs (Table S2). Eed1 was previously shown to be required for growth on the TCA cycle intermediates citrate, lactate, and acetate (79, 80), and the carbon-source-responsive zinc finger transcription factor Adr1 acts downstream or independently of Eed1 to regulate citrate utilization (80). An *EED1* knockout mutant showed decreased damage of oral, hepatic, renal, intestinal, and vaginal epithelial cells (20, 42, 79), with a significantly reduced transcription of *ECE1* (20). However, *EED1* was shown to be dispensable in a systemic murine model (79). This suggests a potential link between alternative carbon source utilization and pathogenicity and indicates that Crk1 may interact with factors such as Eed1 in a given biological context. A previous study has shown that Crk1 interacts with Cdc37 and Sti1, and that Cdc37 is required for Crk1 production (81). Cdc37 and Sti1 are both Hsp90 co-chaperone proteins, and Hsp90 and Gcn2 are known to form a complex *in vitro* and *in vivo* (82, 83). Furthermore, Cdc37 and Sti1 are required for the response of Gcn2 to amino acid starvation (82, 83). This suggests that Crk1 is involved in carbon and amino acid metabolism, potentially through the Gcn2/Gcn4 and Hsp90/Cdc37/Sti1 pathways. In line with this, compared to the wild type, the *CRK1* mutant was more resistant to 3-AT (Fig. 4D and E), an inhibitor of catalase activity (84, 85) and histidine biosynthesis (86–88), imposing amino acid starvation (89). Catalases are involved in detoxifying ROS-initiated H_2_O_2_. Increased growth in the presence of 3-AT could suggest that *CRK1* deletion leads to increased catalase activity. Nonetheless, growth of *crk1*Δ/Δ in the presence of H_2_O_2_ was only slightly reduced in a spot dilution assay (yeast morphology) and was similar to the wild-type strain when added after 8 h of incubation at 37°C and 5% CO_2_ in DMEM (hyphae) and minimal media (yeast/pseudohyphae) (Fig. 4C; Fig. S4A and S6), suggesting that the catalase activity is not increased in *crk1*Δ/Δ. Therefore, the increased growth of *crk1*Δ/Δ in the presence of 3-AT could suggest that *CRK1* deletion facilitates Gcn4 activation to confer 3-AT resistance (89). Our observations led to the hypothesis that Crk1 is a negative regulator of metabolic adaptation in *C. albicans*; thus, *CRK1* deletion leads to higher Gcn4 activity and further metabolic rewiring that allows the utilization of alternative carbon sources.

Hyphae formation and the expression of secreted aspartic proteinase (*SAP*) genes are tightly linked to glucose concentrations and the presence of amino acids (29, 90–92). Of note, our transcriptional data showed that the hyphae-associated *SAP* genes (*SAP4–6*) were significantly upregulated on IECs after 18 h (Table S2). Previous research showed that *CRK1* is either detrimental or a positive driver of filamentation depending on the culture media (33, 40). Furthermore, deletion of *CRK1* led to increased fitness during growth with the alternative carbon sources acetate, glycerol, and sorbitol (33). Our data showed that deletion of *CRK1* caused shorter hyphae in the cell culture media DMEM alone or on intestinal epithelial cells (Fig. 2C; Fig. S2C and D). Our KEGG analysis showed that genes significantly differently expressed in *crk1*Δ/Δ compared to the wild type include genes involved in carbon and amino acid metabolism. For example, the expression of glucose transporter genes (*HGT7–8* on C2BBe1 and *HGT1–2* on plastic) was significantly downregulated at 18 h post-infection (Table S2), and the glucose concentration in the supernatant was significantly higher in *crk1*Δ/Δ compared to the wild type after 5–7 h (Fig. S4B), highlighting the importance of Crk1 in glucose sensing and uptake and subsequent filamentation and damage capacity.

Therefore, we suggest that *CRK1* deletion on *C. albicans* causes changes in metabolic regulation and subsequent effects on growth, hyphal formation, expression of hypha-associated genes (including *ECE1* and *SAP4-6*), and interactions with host cells, which in turn lead to the hyper-damaging phenotype. This is also reinforced by the fact that changes in the metabolic conditions during infection of IECs (addition of glucose, supplementation of certain amino acids) resulted in different outcomes for the host-pathogen interaction, especially in terms of epithelial damage (Fig. 4A and B; Fig. S4D and E).

In conclusion, cytotoxicity of *C. albicans* is not necessarily linked to normal growth. The increased cytotoxicity of *crk1*Δ/Δ cannot be explained by enhanced manifestation of classical virulence traits such as enhanced hyphal formation, adhesion, and hyphal elongation, but rather seems to be the consequence of a combination of cellular effects. This includes increased invasion of (shorter) *crk1*Δ/Δ hyphae into intestinal epithelial cells, metabolic changes, and higher expression of hypha-associated genes such as *ECE1* and *SAP4-6* at late time points during infection, leading to enhanced production of candidalysin and proteases. Several data suggest that Crk1 is involved in the regulation of metabolic processes, in particular carbon and nitrogen metabolism, and that this protein kinase contributes to metabolic homeostasis. Therefore, deletion of *CRK1* leads to misregulation and alterations of metabolic and cellular processes, which in turn may lead to the production and secretion of metabolites or factors that are detrimental to epithelial cells, leading to increased cytotoxicity.

## MATERIALS AND METHODS

### Strains and growth conditions

The *C. albicans* strains used in this study are listed in Table S4. All strains were stored as frozen stocks with 20% glycerol at −80°C, sub-cultured on YPD agar (1% yeast extract, 2% peptone, 2% glucose, and 2% agar) at 30°C for 2 days, and stored at 4°C for up to one month. Strains were routinely grown in 24-well plates (damage screen) or flasks (all other experiments) with YPD liquid at 30°C overnight with shaking at 180 rpm. Plates or cells were washed twice with 1% phosphate-buffered saline (PBS; pH 7.4), counted, and diluted depending on the experiment.

### Generation of revertant strains

A *CRK1*-complemented strain was generated using the *SAT1*-Flipper strategy (93). The *CRK1-*ORF, including the 5′ and 3′ regions, was amplified from genomic DNA of *C. albicans* SC5314 using the primers 5′ CRK1-SacII-comp-fw and 3′ CRK1-NotI-comp-rev and assembled with pSFS2A at *Sac*II/*Not*I. The *CRK1* 3′ region was amplified with the primers 3′ CRK1-XhoI-fw and 3′ KpnI-rev and cloned at *Xho*I/*Kpn*I (Table S4). The cloned sequences in the final plasmid, pSFS2A-*CRK1*-comp, were verified by Sanger sequencing. The *CRK1*-complementation cassette was excised from the plasmid using *Sac*II/*Kpn*I and transformed into *crk1*Δ/Δ (M3110/M3111). Transformants were selected on YPD containing 200 µg/ml nourseothricin. Correct integration into the target locus was confirmed by colony PCR at the cassette junctions. The *SAT1* marker was excised from verified transformants by Flp-recombination.

### Culture of host cells

The intestinal epithelial Caco-2 brush border-expressing 1 cell line (C2BBe1; ATCC, CRL2102) (94), the human intestinal goblet-like cell line (HT29-MTX; ATCC, HTB-38, CLS, Lot Nr. 13B021), the oral cell line TR146 (95), and the kidney epithelial HEK293 subclone HEK293A (Invitrogen) were cultured in Dulbecco’s modified Eagle medium (DMEM; Gibco, Thermo Fisher Scientific) containing 10% fetal bovine serum (FBS; Bio&Sell), 10 µg/mL holotransferrin (HTF; Calbiochem, Merck), and 1% non-essential amino acids (NEAAs; Gibco, Thermo Fisher Scientific). The vaginal cell line A-431 (DSMZ number ACC91) was cultured in Roswell Park Memorial Institute medium (RPMI; Gibco) supplemented with 10% FBS. All epithelial cell lines were kept at 37°C with 5% CO_2_ for up to 20 passages. Human umbilical vein endothelial cells (HUVEC) were cultured in endothelial growth medium (ECM; Promocell) containing penicillin and streptomycin (Gibco, 1:100) at 37°C with 5% CO_2_ for 1 or 2 passages. Cell lines were routinely checked for mycoplasma contaminations using a PCR mycoplasma test kit (Promo Kine) according to manufacturer’s instructions. C2BBe1:HT29-MTX mix was seeded at a 70:30 ratio. Host cells were seeded in transwell inserts (polycarbonate membrane with 5 μm pores, Corning, 2 × 10^4^ cells/well), 96-well TPP tissue culture (TC) plates (Merck, 2 × 10^4^ cells/well), 24-well TPP TC plates (Merck, 1 × 10^5^ cells/well), 6-well TPP TC plates (Merck, 3 × 10^5^ cells/well), and 8-well μ-Slide 8-well ibiTreat (ibidi, 3 × 10^4^ cells/well). Differentiated cells were cultured for 13–15 days for differentiation, with a medium exchange every 2 to 4 days. Undifferentiated cells were cultured at 37°C with 5% CO_2_ until confluent (3 days). All infection experiments were carried out in medium without FBS, HTF, or NEAA.

### Quantification of host cell cytotoxicity

#### LDH activity

Host cells were infected with 2 × 10^4^ cells/well *C. albicans* and incubated at 37°C with 5% CO_2_ in DMEM, RPMI, keratinocyte basal medium (KBM; Lonza), or ECM (62). After 24 h, the host cell damage was quantified by measuring the activity of cytoplasmic LDH (96) in the supernatant using the cytotoxicity detection kit (Roche) according to manufacturer’s instructions and as previously described (63). To assess maximum host damage, 0.25% Triton-X was added for 10 min at 37°C with 5% CO_2_.

#### Compromised cell membrane

Undifferentiated IECs (C2BBe1) cells were infected with 1.6 × 10^4^ cells/well *C. albicans* in DMEM containing SYTOX Green nucleic acid stain (Invitrogen, 0.5 μM) and incubated in the Cell Discoverer 7 (Carl Zeiss Microscopy) at 37°C with 5% CO_2_ and imaged every 45 min. The number of SYTOX Green-positive IECs was quantified in ImageJ (97).

### Filamentation

Differentiated IECs (C2BBe1:HT29-MTX) were infected with *C. albicans* at a final concentration of 1 × 10^4^ cells/well in DMEM and incubated at 37°C with 5% CO_2_. After 6 h, samples were fixed with 4% Histofix at room temperature (RT) and stained with calcofluor white as previously described (14). Images were acquired using a Cell Discoverer 7 microscope (Zeiss), and the hyphal length was quantified in ImageJ. On plastic, 1 × 10^4^ cells/well *C. albicans* were added to DMEM and incubated in the IncuCyte time-lapse microscopy system at 37°C with 5% CO_2_ and imaged after 6 h. The confluency and hyphal length were analyzed with the Sartorius Incucyte 2024B Software. Since the hyphal length is affected by the confluency of cells, the hyphal length was divided by the difference of each replicate to the average confluency of each individual strain of the biological triplicates.

### Fungal growth

*C. albicans* was added to YPD or synthetic defined medium (SD; 0.17% yeast nitrogen base [YNB] without amino acids (AA) and ammonium sulfate [AS], 0.5% AS, 0.4% or 2% glucose, pH 6.0) at a final concentration of 2 × 10^4^ cells/well. Plates were incubated in a Tecan infinite M200 pro reader at 30°C. The growth of *C. albicans* in the presence of inhibitors was assessed in YPD (2% glucose) in the presence of 7.5 nM rapamycin or in YPD (2% glucose) and SD (0.4% or 2% glucose) in the presence or absence of 3 μM antimycin A, 10 μM oligomycin, 20 μM CCCP, or 100 μM 3-AT. The absorbance (OD_600_) was measured every 30 min for 50 or 72 h. For analysis, the uninfected media control and the baseline OD_600_ at 0 h were subtracted. Different growth stages were calculated in GraphPad Prism 10.5.0. For hyphae growth, 1 × 10^4^ cells/well *C. albicans* were added to YPD and SD and incubated in the IncuCyte time-lapse microscopy at 37°C with 5% CO_2_. Images were acquired every 30 min over 24 h. The confluency of *C. albicans* was analyzed with the Sartorius Incucyte 2024B Software.

### Growth on TCA cycle intermediates

*C. albicans* was adjusted to an OD_600_ of 0.2/mL in 25 mL of YNB media (0.17% YNB without AA, AS, and biotin; 0.5% AS; 58 mM glucose, sodium lactate, pyruvate, citrate, succinate, malate, or α-ketoglutarate; pH 5.0) and incubated at 30°C and 180 rpm for 48 h. The OD_600_ was measured after 0, 6, 12, 24, and 48 h.

### Adhesion and invasion

Undifferentiated IECs (C2BBe1) were infected with 1 × 10^5^ (adhesion, 24-well plate) or 1.5 × 10^4^ (invasion, ibidi) cells/well *C. albicans* and incubated at 37°C with 5% CO_2_. After 1 (adhesion) or 8 h (invasion), plates were washed and fixed with 4% Histofix for 15 min at RT and stained as previously described (14). Images were acquired with the Zeiss Cell Discoverer 7 (adhesion) or Zeiss AXIO Observer.Z1 (invasion). The number of adhered fungal cells was counted using CellProfiler 4.2.8 (98) (Fig. S7) and averaged across 100 images (650 × 520 µm) per biological replicate. The percentage of invasive hyphae was calculated from the count of extracellular and all (external + internal) hyphae and averaged from 10 images per biological replicate. The percentage of intracellular fungal mass was measured using the graphical programming language JIPipe (99) (Fig. S8 and S9) applied on images after deconvolution with Huygens Professional 25.10.0p2. The formation of transcellular tunnels by *C. albicans* in Caco-2 cells in phenol red-free DMEM (Gibco) was assessed as previously described (61). Transcellular tunnels were counted using ImageJ and are relative to the number of inoculating yeasts. Invasion into YNB + GlcNAc agar plates (0.17% YNB without AA, AS, 0.5% AS, 2% N-acetylglucosamine [GlcNAc], 1.5%, 2%, 3%, or 4% agar, pH 6.0) was assessed as previously described (100). Images were acquired after 2, 3, and 4 days after plates were washed with dH_2_O to remove non-adhered cells.

### Candidalysin in the invasion pocket

Undifferentiated IECs (C2BBe1) were infected with 7.5 × 10^3^ cells/well *C. albicans* and incubated at 37°C with 5% CO_2_ for 8 h. *C. albicans* candidalysin was stained as previously described (20) with the difference that 0.5% Triton was used to permeabilize cells (described in invasion).

### Translocation and epithelial barrier integrity

Differentiated IECs (C2BBe1:HT29-MTX) were infected with 2 × 10^4^ cells/well *C. albicans*, and the translocation (after 16 and 24 h), zymolyase sensitivity, and resistance were assessed as previously described (14).

### Glucose uptake

*C. albicans* was adjusted to an optical density (OD_600_) of 0.2/ml in 35 mL of YNB media (0.17% YNB without AA, AS, and biotin, 0.5% AS, 20 mM glucose) and incubated at 37°C and 180 rpm. The glucose concentration in the supernatant was measured hourly, starting after 5 h using the Freestyle Precision Neo measuring system until it fell below the detection limit (20 mg/dL). The glucose concentration of KBM medium was tested using the Freestyle Precision Neo measuring system.

### RNA-seq

*C. albicans* wase added to differentiated C2BBe1 or 6-well plates at a final concentration of 2 × 10^6^ cells/well and incubated at 37°C with 5% CO_2_. After 6 and 18 h, plates were washed once with ice-cold PBS, and 650 μL of RLT buffer (RNeasy Minikit, Qiagen) supplemented with β-mercaptoethanol (1:100) was added to each well. The plates were frozen with liquid nitrogen, and the wells were scraped. The host (supernatant) and fungal (pellet) RNA were separated. The host RNA was isolated using the RNeasy Minikit according to the manufacturer’s instructions. The fungal RNA was isolated using the freeze-thaw method as previously described (15).

Pre-processing of raw reads, including quality control and gene abundance estimation, was performed using the GEO2RNaseq pipeline (101) in R (version 4.4.3). Quality analysis was done with FastQC (v0.11.8) before and after trimming. Read quality trimming was performed using Trimmomatic (v0.36). Reads were rRNA-filtered using SortMeRNA (v2.1) with a single rRNA database combining all rRNA databases shipped with SortMeRNA. The combined reference genome in FASTA format was created by combining the references for *Homo sapiens* (GRCh38, version 109) and *Candida albicans* (SC5314 version A22s07-m01-r100). Reference annotation was created by extracting and combining exon features from the corresponding annotation files. The combined reference genome was indexed with exon information using HiSat2 (v2.1.0, paired-end mode). Gene abundance estimation was performed using featureCounts (v1.28.0) in paired-end mode with default parameters. MultiQC version 1.7 was finally used to summarize and assess the quality of the output of FastQC, Trimmomatic, HiSat, featureCounts, and SAMtools. The count matrix with gene abundance data without and with median-of-ratios normalization (MRN) (102) were extracted. Raw files will be accessible upon acceptance of the manuscript by the BioStudies ArrayExpress collection. Differential gene expression for pairwise tests was analyzed using two statistical tools (DESeq2 v1.18.1 and edgeR v3.20.7) to report raw and multiple-testing-corrected *P* values using the false discovery rate method, *q* = FDR(p), for each tool. In addition, mean MRN, transcripts per kilobase million (TPKM), and reads per kilobase million (RPKM) values were computed per test per group, including corresponding log2 fold changes. Gene expression differences were considered significant if they were reported significant by both tools (*q* ≤ 0.05 and |log2 (MRN)| ≤1). KEGG enrichment analysis was carried out as previously described (103).

### Biolog phenotypic microarray

*C. albicans* was pre-grown on YPD agar plates at 30°C for 2 days. Cells were prepared and added to Biolog Phenotypic Microarrays for microbial cells (PM1–3, 6–8) according to the manufacturer’s protocol for yeasts (Biolog, Inc., Hayward, CA, USA). The AUC was calculated in GraphPad Prism 10.5.0, and the negative control from each plate was subtracted. Substrates on which the strains grew with a difference of less than AUC 50 to the negative control were labeled as not growing. A heatmap displaying the AUCs was created using the pheatmap function in R-4.4.2.

### Stress test

*C. albicans* (10^7^ cells/mL) were 10-fold serially diluted, and 5 µL was spotted onto a YPD with or without either 1 M sodium chloride (NaCl); 0.6 M potassium chloride (KCl); 1, 2.5, or 5 mM *tert*-butyl hydroperoxide (t-BOOH); 2.5 or 5 mM hydrogen peroxide (H_2_O_2_); 10 or 20 μg/mL calcofluor white; 50, 100, or 150 μg/mL Congo red; 5, 10, or 15 μg/mL caffeine; or 0.1 or 0.2% sodium lauryl sulfate (SDS), and incubated at 30°C, 37°C, or 42°C for 2 days. The growth of each strain was rated qualitatively on a scale of −3 to 3 compared to the wild type on the same growth condition (0 equals similar, −3 to −0.5 equals reduced, and 0.5 to 3 equals increased growth). To test stress in hyphae, 5 × 10^3^ cell/well *C. albicans* was added to DMEM or SD (2% glucose) and incubated at 37°C with 5% CO_2_. After 8 h, 0.5 or 1 mM of H_2_O_2_ was added, and the cells were incubated in the IncuCyte time-lapse microscopy at 37°C with 5% CO_2_ and imaged every 30 min over 16 h. The confluency of *C. albicans* was analyzed using the Sartorius IncuCyte 2024B Software.

### Statistical analysis

Data were analyzed using GraphPad Prism version 10 (GraphPad Software, La Jolla, California, USA) unless otherwise stated, and the statistical significance is depicted in the figures as *P* value. Data from at least three biological and technical replicates were analyzed and values are depicted as mean or percentage ± standard deviation. All microscopy and stress test analyses were carried out in three biological replicates, and representative images are provided in the manuscript.

## SUPPLEMENTAL MATERIAL

Supplemental figures mbio.01764-26-s0001.pdf Fig. S1 to S9.

Table S1 mbio.01764-26-s0002.xlsx Expression of C2BBe1 genes infected with *crk1*Δ/Δ deletion mutant vs wild type.

Table S2 mbio.01764-26-s0003.xlsx Expression of *C. albicans* genes in *crk1*Δ/Δ deletion mutant vs wild type.

Table S3 mbio.01764-26-s0004.xlsx Results of Biolog phenotypic microarray carbon plates.

Table S4 mbio.01764-26-s0005.xlsx Strains and primers used in this study.

ASM does not own the copyrights to Supplemental Material that may be linked to, or accessed through, an article. The authors have granted ASM a non-exclusive, world-wide license to publish the Supplemental Material files. Please contact the corresponding author directly for reuse.
