# Supplementary material for: Functional analysis of Candida albicans protein kinases identifies Crk1 as a modulator of epithelial cell damage
Source: mBio. 2026 Aug 20;17(9):e01764-26. doi: 10.1128/mbio.01764-26 (PMC13556258; doi:10.1128/mbio.01764-26)
Supplement: Supplementary file 1 [file mbio.01764-26-s0001.pdf]

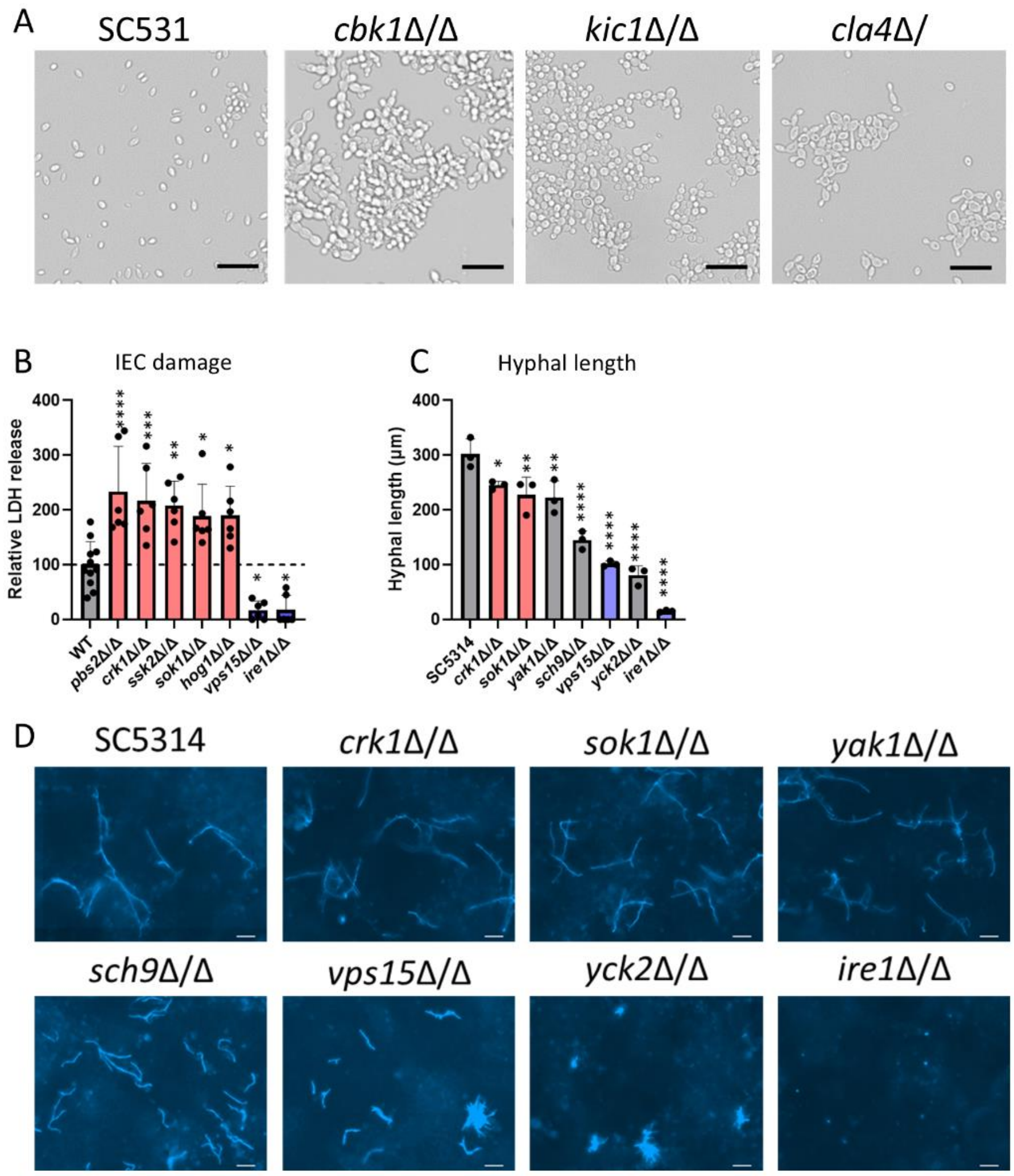

Fig. S1. Protein kinases involved in IEC damage and filamentation. **A** Mutants causing cell aggregation were excluded from further experiments. Scale bar represents 20 µm. **B** Mutants with significantly increased or decreased intestinal epithelial cell (IEC) damage and are displayed as percentage to the wild type. Data represents measuring the release of cytoplasmic lactate dehydrogenase (LDH) from 6 biological and 3 technical replicates (cytotoxicity screen + validation). **C** Mutants with significantly reduced hyphal length in DMEM medium on IECs at 6h post infection at 37°C and 5% CO_2. Mutants with significantly increased cytotoxicity (**B**) are highlighted in red and significantly decreased cytotoxicity (**B**) in blue. **D** Representative images for measuring hyphal length. *C. albicans* is stained with calcofluor white and the scale bar represents 50 µm. Bars represent the mean ± standard deviation and

dots represent the mean of each biological replicated. Data were tested for significance using one-way ANOVA and Dunnett's multiple comparisons test (**B-C**). * $p < 0.05$, ** $p < 0.01$, *** $p < 0.001$, **** $p < 0.0001$

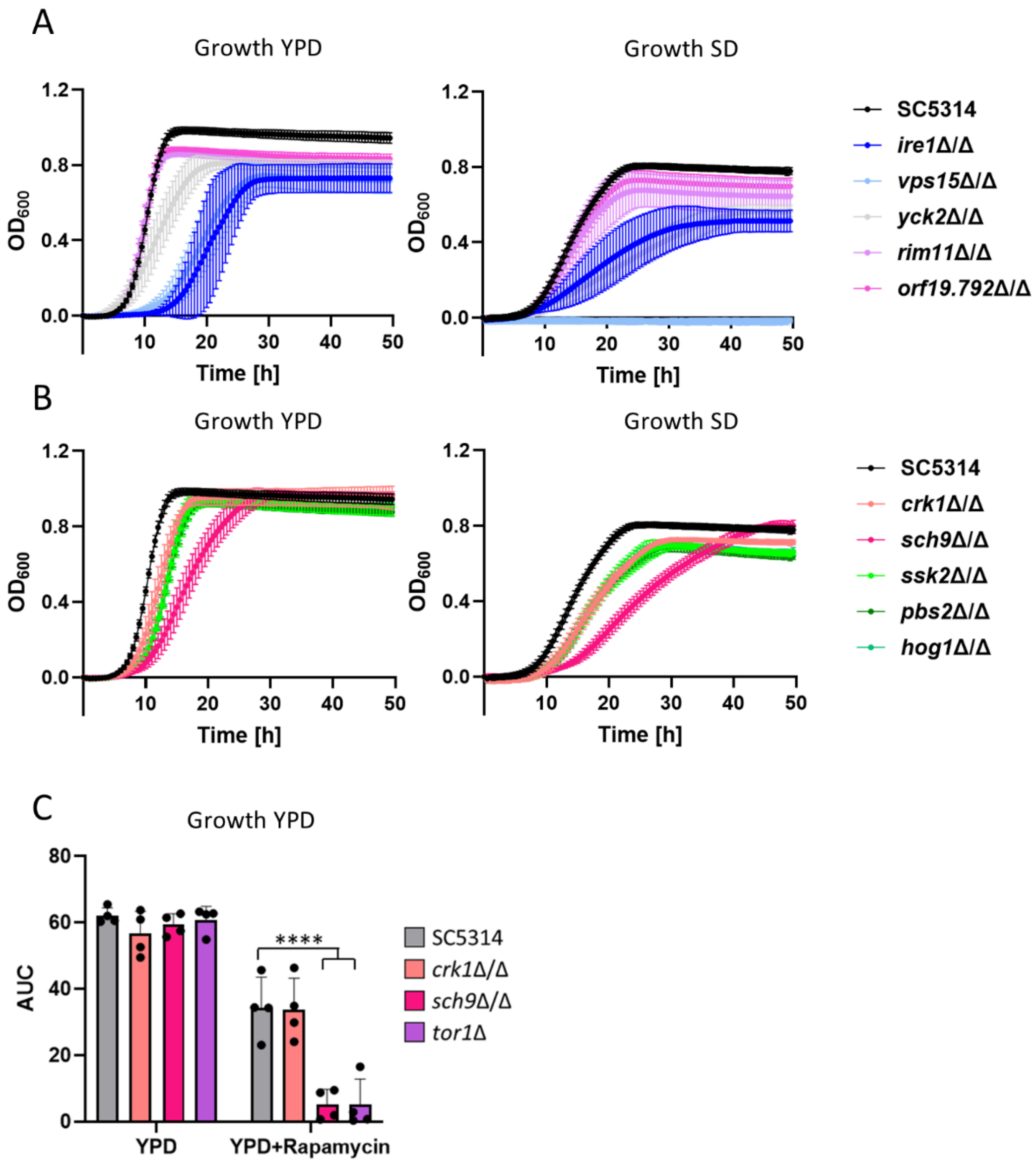

Fig. S2. Protein kinases involved in growth. Mutants displaying reduced (**A**) or slower (**B**) growth compared to the wild type in rich (YPD) or minimal (SD) media. Error bars represent the mean ± standard deviation. **C** Area under the curve (AUC) of growth curves in the presence or absence of 7.5 nM rapamycin. Bars represent the mean ± standard deviation and dots represent the mean of each biological replicated. Data were tested for significance using two-way ANOVA and Dunnett's multiple comparisons test. **** $p < 0.0001$

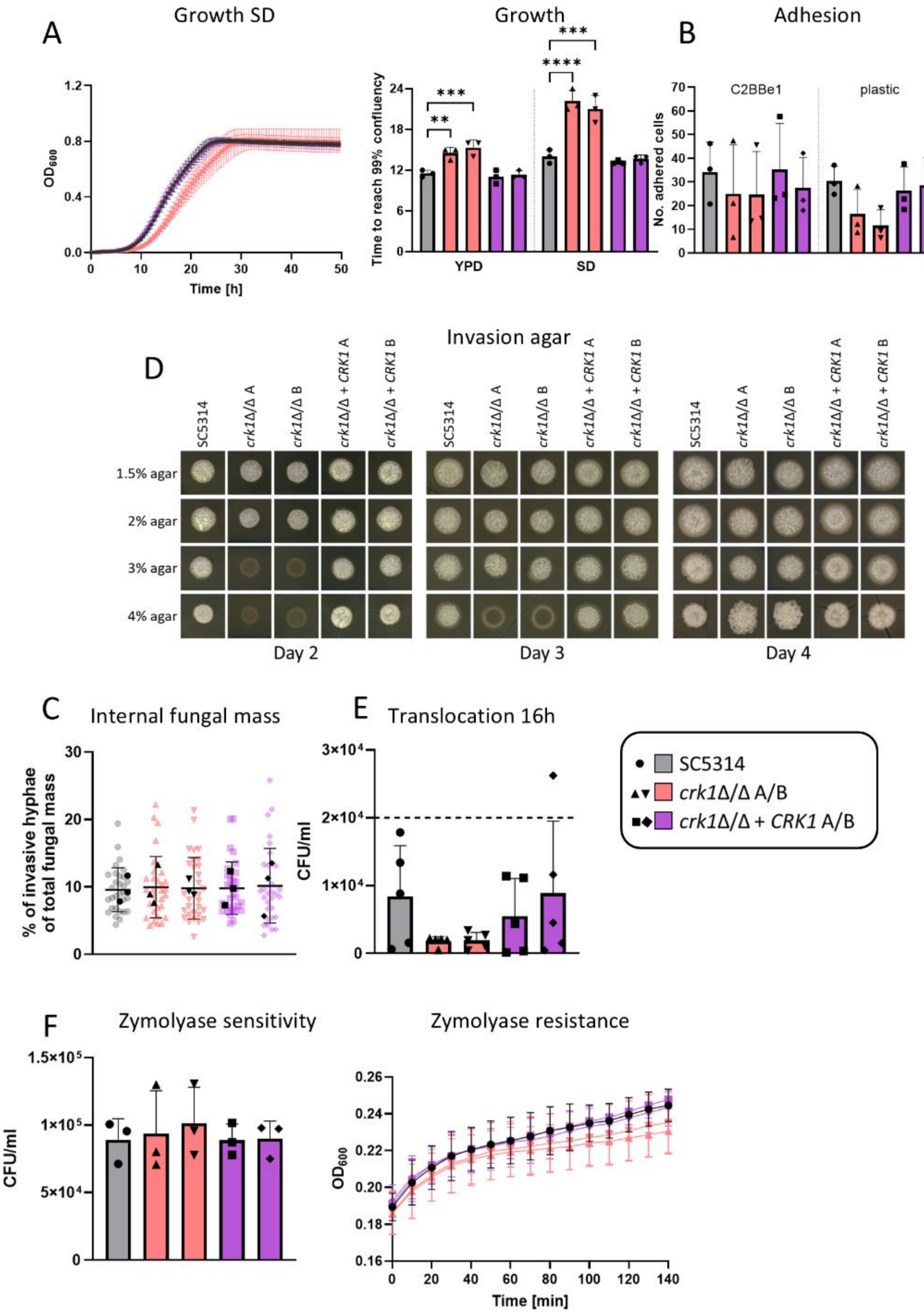

Fig. S3. Crk1 is required for agar invasion and translocation. A Growth in minimal media was measured by measuring the OD_{600} every 30 minutes over 50 h at 30°C or for 24h at 37°C. Error bars represent the mean ± standard deviation. To measure growth at 37°C and 5% CO_2, the confluency in each field of view was analysed and the time to reach 99% confluency was calculated. **B** The number of adhered *C. albicans* yeast cells to C2BBe1 or plastic after 1h was counted. **C** Internal fungal mass was calculated by measuring the invasive hyphae parts relative to all hyphae (external + internal) per field of view at 8h post infection. **D** *C. albicans*

strains were spotted on plates containing N-acetylglucosamine (GlcNAc) and images were acquired after washing with dH_2O after 2, 3 and 4 days at 37°C **E** The colony forming units (CFUs) per ml were counted and calculated after 16h relative to the initial inoculum. The dotted line represents the initial inoculation. **F** After 3h of incubation at 37°C, 20 U/ml zymolyase was added, followed by another 2h incubation at 37°C. To measure zymolyase sensitivity, the colony forming unit (CFU) per ml was counted and calculated. To measure zymolyase resistance, 20 U/ml were added after the initial 3 hours incubation and the OD_{600} was measured every 10 minutes for 140 minutes. Bars represent the mean ± standard deviation and dots represent the mean of each biological replicated. Data were tested for significance using one-way ANOVA and Dunnett's multiple comparisons test (**A-B, C-F**). Statistical test was carried out separately for C2BBe1 and plastic adhesion (**B**) ** $p < 0.01$, *** $p < 0.001$, **** $p < 0.0001$

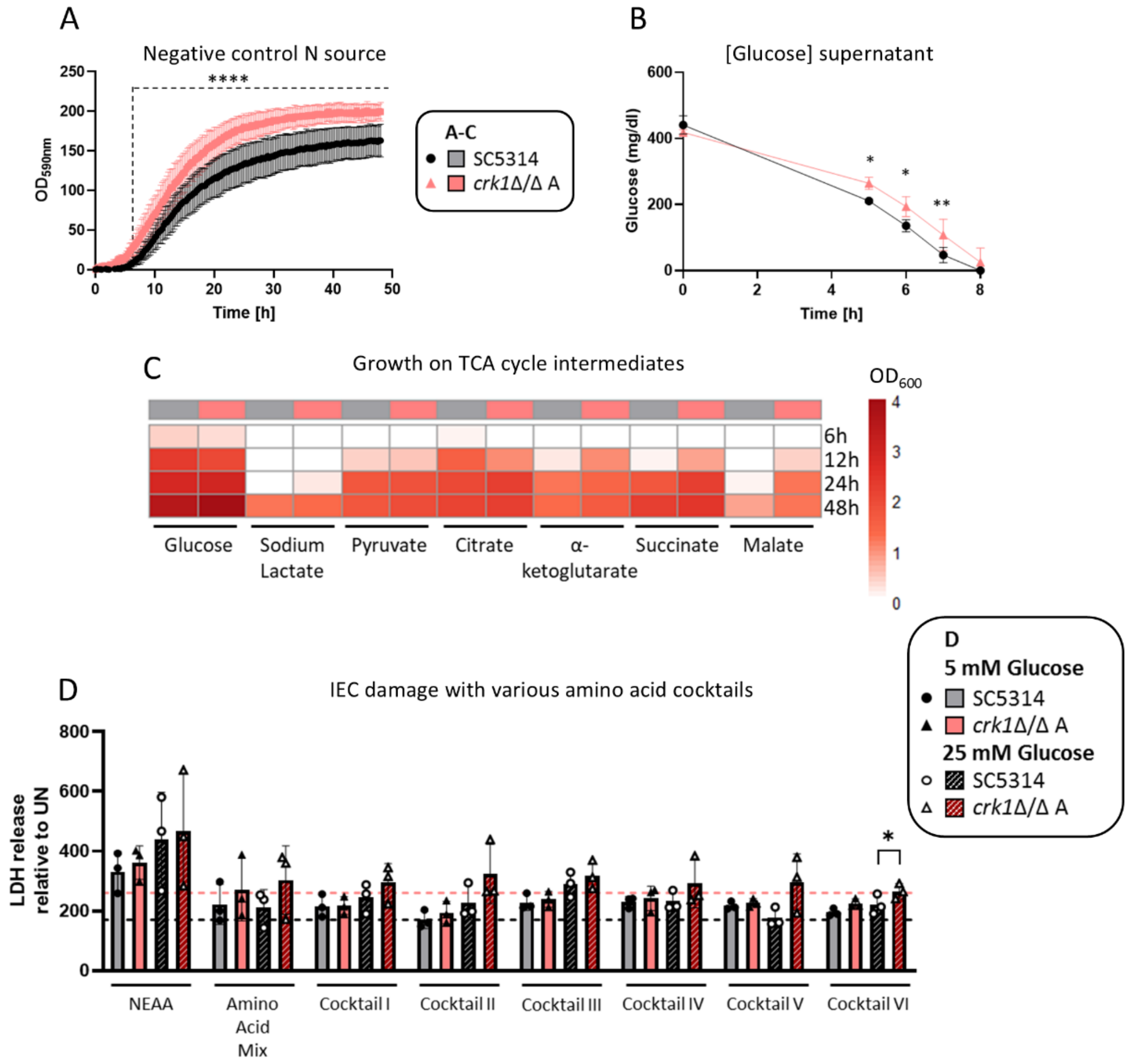

Fig. S4. Loss of *CRK1* increases growth on TCA cycle intermediates. A The metabolic activity of wild type (SC5314) and *crk1*Δ/Δ in the negative control of the Biolog phenotypic microarray nitrogen plates. Data were tested for significance using two-way ANOVA and Šídák's multiple comparisons test. Error bars represent the mean ± standard deviation. **B** Glucose concentration in minimal media (containing 20 mM Glucose). The glucose concentration was measured in the supernatant of wild type and *crk1*Δ/Δ. Data were tested for significance using two-way ANOVA and Šídák's multiple comparisons test. Error bars represent the mean ± standard deviation. **C** The growth ($OD_{600\ nm}$) of wild type and *crk1*Δ/Δ was measured after 6, 12, 24, 48h in YNB with 58 mM glucose, sodium lactate, pyruvate, citrate, α-ketoglutarate, succinate or malate as the sole carbon source. **D** LDH release relative to the uninfected (UN)

control. IECs were infected with wild type (SC5314) or *crk1*Δ/Δ with EM media containing different amino acids cocktails. The dotted lines represent the LDH release of wild type (black) and *crk1*Δ/Δ (red) in DMEM. Bars represent the mean ± standard deviation and dots represent the mean of each biological replicated. Data were tested for significance using two-way ANOVA and Tukey's multiple comparisons test * $p < 0.05$, ** $p < 0.01$, **** $p < 0.0001$

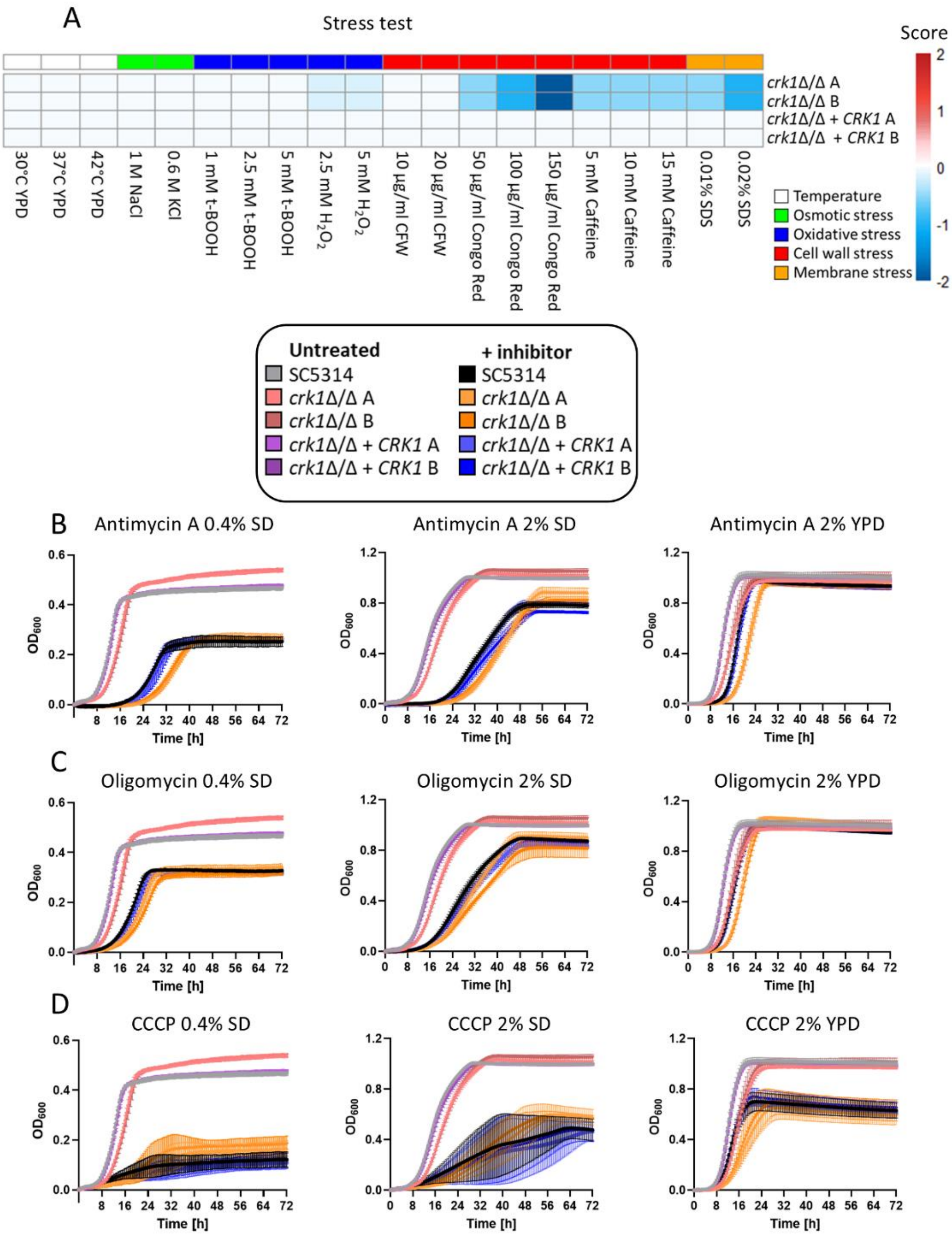

Fig. S5. Crk1 is dispensable for growth on mitochondria inhibitor but required for cell wall stress response. A The heatmap shows the fitness score of *crk1*Δ/Δ A/B and *crk1*Δ/Δ + *CRK1* A/B relative to the wild type in the presence of temperature, osmotic, oxidative, cell wall or membrane stressors. **B-D** Growth of *C. albicans* strains in minimal (0.4% or 2% glucose) or rich (2% glucose) media in the presence of 3 µM Antimycin A (**B**) or 10 µM Oligomycin (**C**) or 20 µM CCCP (**D**). Error bars represent the mean ± standard deviation.

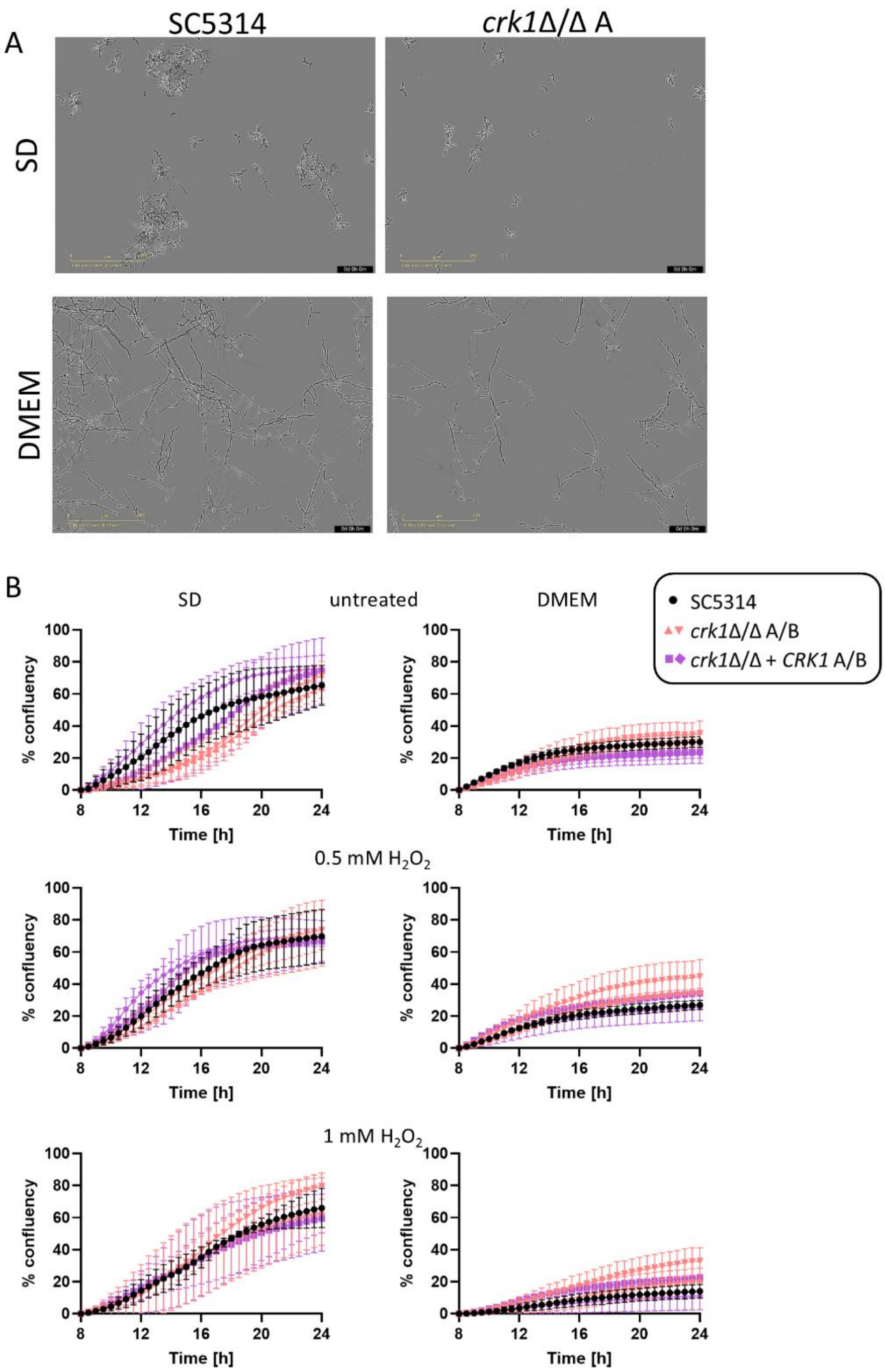

Fig. S6. Response of hyphae to oxidative stress. A Respective images for the morphology of *C. albicans* strains after 8h incubation at 37°C with 5% CO_2 in minimal (SD, 2% glucose) or cell culture media (DMEM). **B** After 8h incubation at 37°C with 5% CO_2, 0, 0.5 or 1 mM H_2O_2 was added and the growth was assessed in minimal or DMEM at 37°C with 5% CO_2. Images were acquired hourly over 16 hours and the confluency was analysed. Error bars represent the mean ± standard deviation.

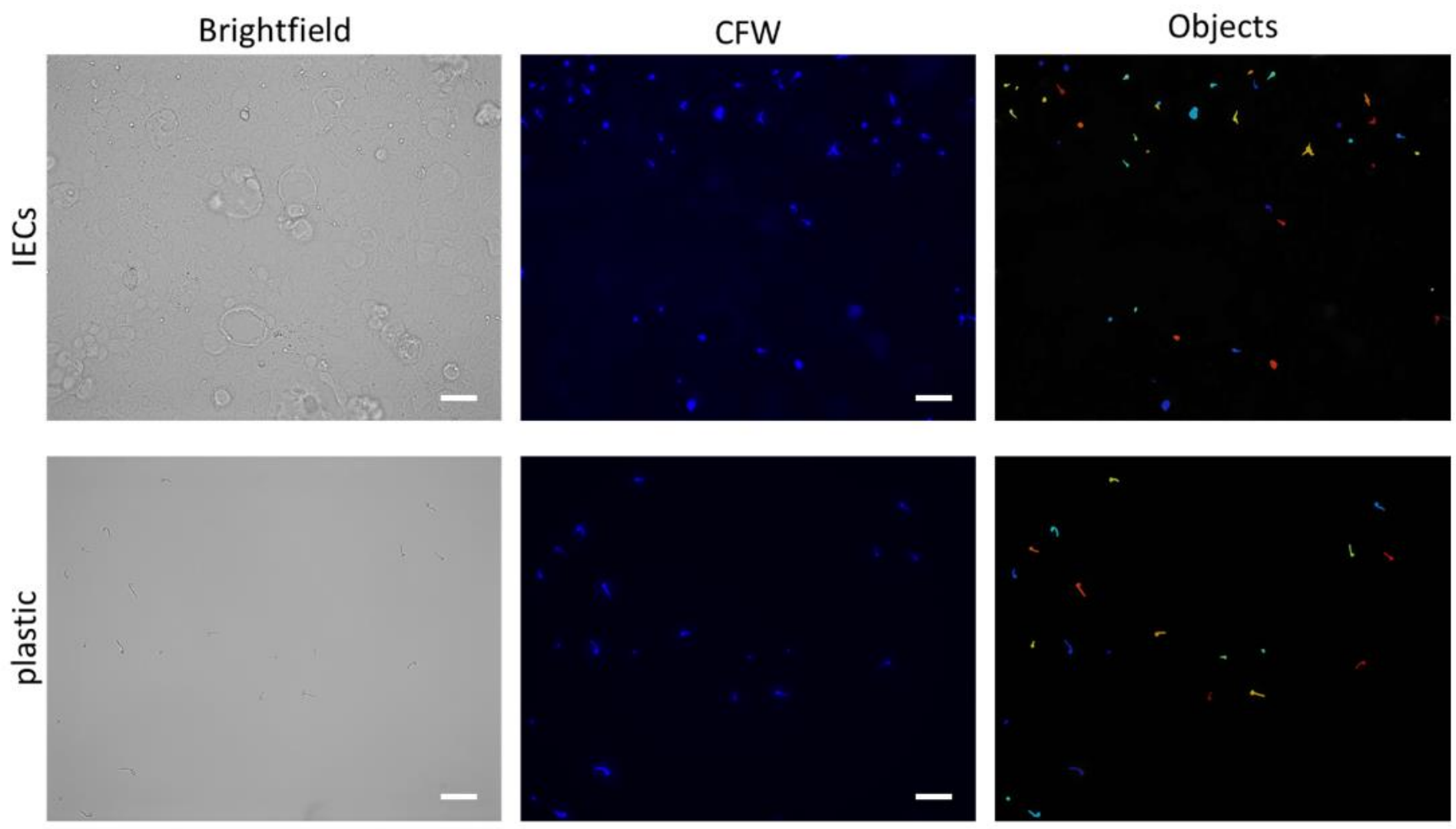

Fig. S7. Adhesion image analysis pipeline in Cellprofiler 4.2.8. The input image was the calcofluor white (CFW) stained. *C. albicans* strains on IECs (top) were thresholded by the global strategy Otsu method with the lower bounds on threshold at 0.04. Shape was used to distinguish and draw dividing lines between clumped objects. Images of *C. albicans* on plastic were first enhanced with the enhancement method tubeness with a smoothing scale of 4. The enhanced image was then thresholded by the global strategy and Minimum Cross-Entropy method with the lower bounds on threshold at 0.001. Shape was used to distinguish and intensity to draw dividing lines between clumped objects.

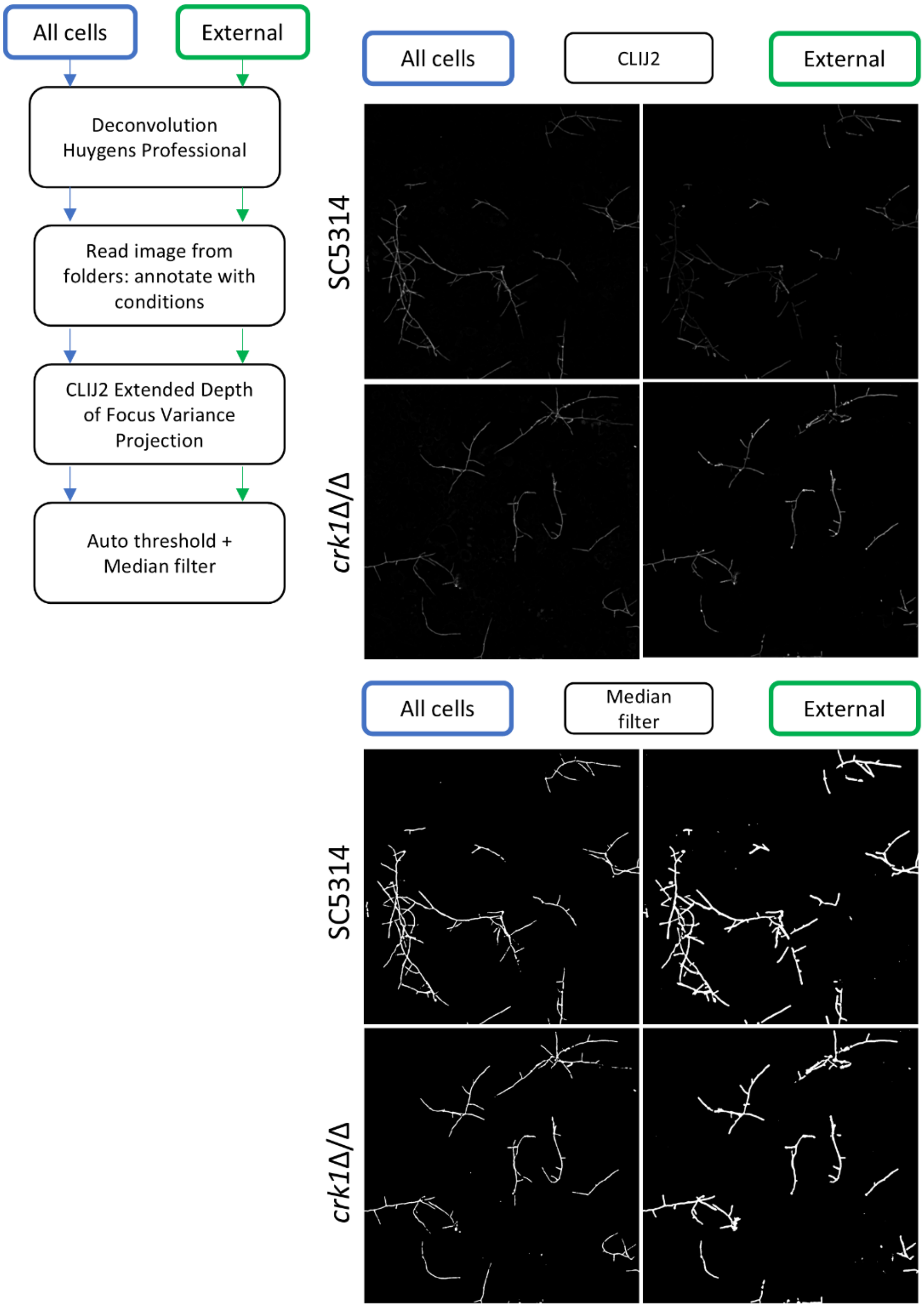

Fig. S8. Invasion image analysis pipeline in JIPipe 5.3.0 and Huygens Professional 25.10.0p2 for image preparation. Images were acquired with Z stacks and then deconvoluted in Huygens. Then images were read into JIPipe and were annotated with conditions. 3D Images were changed into 2D images by using CLIJ2 extended depth of focus variance projection (top images) and then auto thresholded using the Otsu (all cells) or Huang (external) method and a median filter was applied (bottom images).

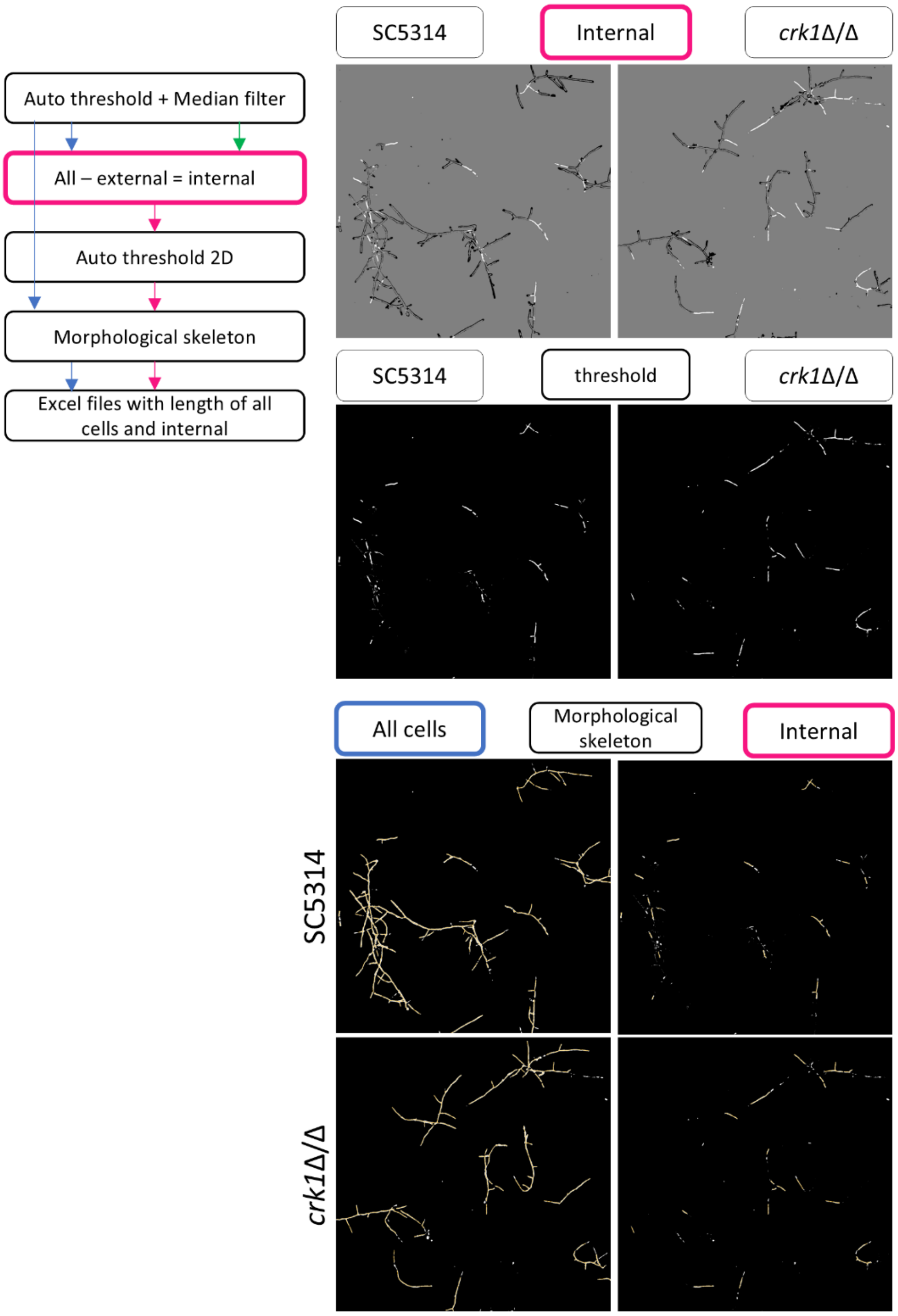

Fig. S9. Invasion image analysis pipeline in JIPipe 5.3.0 to measure fungal length. Following thresholding and median filter (**Fig. S8**), the external image was subtracted from the entire (all) image (top) and the new internal image was thresholded using the default method. From the internal and all cells images, morphological operation and morphological skeletonize was used to identify filaments with a radius bigger or equal to 1 and length in pixels of more than

20. These measurements were then exported into excel and the percentage of invasive hyphal parts relative to all cells was calculated.
